# Prepubertal skeletal muscle growth requires Pax7-expressing satellite cell-derived myonuclear contribution

**DOI:** 10.1242/dev.167197

**Published:** 2018-10-25

**Authors:** John F. Bachman, Alanna Klose, Wenxuan Liu, Nicole D. Paris, Roméo S. Blanc, Melissa Schmalz, Emma Knapp, Joe V. Chakkalakal

**Affiliations:** 1 Department of Pathology and Laboratory Medicine, Cell Biology of Disease Graduate Program, University of Rochester Medical Center, 601 Elmwood Ave, Rochester, NY 14642, USA; 2 Department of Pharmacology and Physiology, University of Rochester Medical Center, 601 Elmwood Ave Box 711, Rochester, NY 14642, USA; 3 Department of Biomedical Genetics, Genetics, Development, and Stem Cells Graduate Program, University of Rochester Medical Center, 601 Elmwood Ave Box 633, Rochester, NY 14642, USA; 4 Department of Orthopaedics and Rehabilitation, Center for Musculoskeletal Research, University of Rochester Medical Center, 601 Elmwood Ave, Rochester, NY 14642, USA; 5 Wilmot Cancer Institute, Stem Cell and Regenerative Medicine Institute, The Rochester Aging Research Center, and Department of Biomedical Engineering, University of Rochester Medical Center, 601 Elmwood Ave, Rochester, NY 14642, USA

**Keywords:** Aging, Extracellular matrix, Hypertrophy, Mouse, Musculoskeletal, Pediatric, Postnatal growth, Regeneration, Stem cell

## Abstract

The functional role of Pax7-expressing satellite cells (SCs) in postnatal skeletal muscle development beyond weaning remains obscure. Therefore, the relevance of SCs during prepubertal growth, a period after weaning but prior to the onset of puberty, has not been examined. Here, we have characterized mouse skeletal muscle growth during prepuberty and found significant increases in myofiber cross-sectional area that correlated with SC-derived myonuclear number. Remarkably, genome-wide RNA-sequencing analysis established that post-weaning juvenile and early adolescent skeletal muscle have markedly different gene expression signatures. These distinctions are consistent with extensive skeletal muscle maturation during this essential, albeit brief, developmental phase. Indelible labeling of SCs with *Pax7^CreERT2/+^*; *Rosa26^nTnG/+^* mice demonstrated SC-derived myonuclear contribution during prepuberty, with a substantial reduction at puberty onset. Prepubertal depletion of SCs in *Pax7^CreERT2/+^*; *Rosa26^DTA/+^* mice reduced myofiber size and myonuclear number, and caused force generation deficits to a similar extent in both fast and slow-contracting muscles. Collectively, these data demonstrate SC-derived myonuclear accretion as a cellular mechanism that contributes to prepubertal hypertrophic skeletal muscle growth.

## Abstract (precis)

**Summary:** Examination of gene expression and morphological changes in mouse skeletal muscle during prepuberty demonstrates that satellite cell-derived myonuclear accretion contributes to prepubertal hypertrophic skeletal muscle growth.

## INTRODUCTION

Prepuberty is a period of pediatric growth that precedes early adolescence (Dutta and Sengupta, 2016). After weaning and prior to the onset of puberty, with the induction of sex hormones marking the beginning of adolescence, skeletal muscle undergoes extensive growth and maturation (Ober et al., 2008; Schiaffino et al., 2013). Skeletal muscles are the primary effectors of force production and movement. They are composed of a heterogeneous mixture of multinucleated muscle fibers (myofibers), which vary in size and/or contractile ability (Schiaffino and Reggiani, 2011). Although notable changes in skeletal muscle morphology occur during prepubertal growth, myofiber number does not increase, as this is established in the late embryonic-early postnatal period (Ontell et al., 1984; White et al., 2010). Postnatal skeletal muscle growth primarily occurs through expansion of myofiber cross-sectional area (CSA) and length (Agbulut et al., 2003; White et al., 2010). This period is also characterized by myonuclear accretion, the addition of myonuclei. Myonuclear accretion is thought to cease at weaning age in some muscles (White et al., 2010). Therefore, post-weaning myofiber growth is deemed to be regulated primarily through post-mitotic mechanisms. However, whether myonuclear accretion continues and the relevance of this process to prepubertal myofiber growth has yet to be examined.

Each myofiber is endowed with a pool of resident stem cells, termed satellite cells (SCs). SCs are the derivatives of dermomyotome-derived myogenic progenitors, responsible for initial embryonic and early postnatal skeletal muscle development (Chal and Pourquie, 2017; Gros et al., 2005; Kassar-Duchossoy et al., 2005; Relaix et al., 2005). As development proceeds, populations of cells expressing the paired box transcription factor Pax7 escape terminal myogenic commitment. These cells are set aside to serve as a reserve source of myonuclei in response to growth, injury and adaptation (Buckingham and Relaix, 2007; Tajbakhsh, 2009). In adult skeletal muscle, SCs reside at the interface between the myofiber and basal lamina, primarily in a quiescent state. However, in response to degenerative myofiber injury, denervation, or other triggers, SCs can be activated to participate in programs of repair or regeneration (Klose et al., 2018; Liu et al., 2017, 2015; Paris et al., 2016; Sambasivan et al., 2011). Various groups, through the use of either specific depletion of Pax7-expressing SCs or disruption of Pax7 expression, have demonstrated the indispensable nature of SCs in myofiber regeneration, repair and maintenance (Egner et al., 2016; Fry et al., 2014; Gunther et al., 2013; Klose et al., 2018; Lepper et al., 2011; Liu et al., 2017, 2015; Murphy et al., 2011; Relaix and Zammit, 2012; von Maltzahn et al., 2013).

Late embryonic and early postnatal skeletal muscle growth is characterized by extensive SC activity. In contrast, SC number drastically declines between early postnatal and weaning age (Dayanidhi and Lieber, 2014; Neal et al., 2012; White et al., 2010). However, labeling with non-quiescent and myogenic progenitor markers indicates continued SC activity up until the onset of adolescence (Chakkalakal et al., 2014; Kim et al., 2016). In addition, cycling SCs more prone to terminal commitment have been identified at various stages throughout postnatal life (Chakkalakal et al., 2014, 2012a; Schultz, 1996). These data suggest a subpopulation of SCs may continue to contribute to postnatal myofiber growth and maintenance. Indelible labeling of Pax7-expressing SCs with a cytoplasmic fluorescent reporter, 2 weeks prior to the examination of fate, indicates that SC-derived contributions to myofibers plateau at a young adult stage (8-12 weeks) (Pawlikowski et al., 2015). However, whether such contributions reflect myonuclear turnover and/or accretion has yet to be elucidated. Similar labeling strategies have also demonstrated SC-derived contributions to aging myofibers (Keefe et al., 2015; Liu et al., 2017; Tierney et al., 2018).

The role of Pax7-expressing myogenic cells in embryonic development has proven to be essential; their absence results in severe and immediate deficits in the formation of fetal myofibers (Hutcheson et al., 2009). In contrast, SC depletion in sedentary adult mice leads to myofiber atrophy in some, but not all, muscles, and this manifests over a relatively protracted period of time (Fry et al., 2015; Keefe et al., 2015; Liu et al., 2017). Despite evidence supporting SC contribution and activity during prepuberty, there have yet to be any studies elucidating the immediate impact of SC-derived progenitor loss on prepubertal myofiber size, myonuclear number and skeletal muscle function. Therefore, post-weaning skeletal muscle growth and maturation is thought to be independent of any immediate and significant contribution from SC-derived myonuclear accretion (Relaix and Zammit, 2012; Stantzou et al., 2017; White et al., 2010).

In this study, we investigated skeletal muscle development during prepuberty and examined the consequences of diminished SC contribution to this maturation. We characterized myofiber CSA, myonuclear number, and SC number from prepuberty to early adult stages for fast-contracting extensor digitorum longus (EDL) and slow-contracting soleus (SOL) muscles. Quantification of myonuclear number revealed continuous myonuclear accretion up to 6 weeks of age (puberty onset/early adolescence). Additionally, through RNA sequencing (RNAseq) we identified significant changes in gene expression, pertinent to skeletal muscle maturation, occurring between 4 and 6 weeks. The most notable changes were related to calcium handling, AMPK signaling and extracellular matrix (ECM) content. Lineage tracing of SCs revealed significant myonuclear contribution during prepuberty, with considerable drop-off at the onset of puberty. Prepubertal SC depletion, as opposed to during early adolescence, led to a significant decline in the intrinsic force-generation capacity of skeletal muscles. This decline in force generation was associated with similar reductions in myofiber CSA and myonuclear content in both EDL and SOL muscles. Collectively, these data reveal that specific loss of Pax7-expressing SCs and derived myonuclei during prepuberty, a crucial time of whole organism development, is sufficient to induce immediate deficiencies in skeletal muscle growth and maturation.

## RESULTS

### Extensive myofiber hypertrophy during prepuberty and early adolescence

**Fig. 1.**
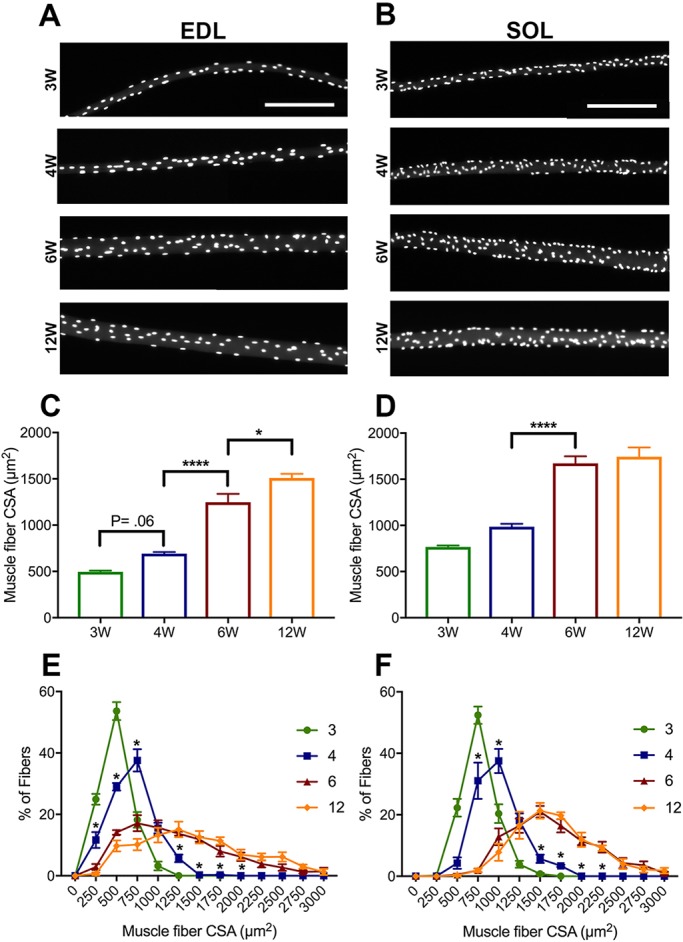
**Myofibers increase in size during prepubertal growth, with an adult-like population established at 6 weeks.** (A,B) Representative images of EDL (A) and SOL (B) 3-, 4-, 6- and 12-week of age myofibers. Scale bars: 200 μm. (C,D) Bar graphs comparing the CSA of EDL (C) and SOL (D) 3-, 4-, 6- and 12-week myofibers. *n*=5 mice per group. *P<0.05, ****P<0.0001 (one-way ANOVA with Tukey's test). (E,F) Frequency distribution of myofiber CSA in EDL (E) and SOL (F). Two-way ANOVA with Tukey's test. *P<0.05 (significance between 4 and 6 weeks). Statistical comparisons are provided in Table** **S1.

In order to assess mouse skeletal muscle growth during prepuberty, adolescence and young adulthood, the myofiber CSA from C57BL/6 EDL and SOL muscles was measured. The EDL and SOL are representative skeletal muscles composed primarily of faster and slower contracting myofibers, respectively (Schiaffino and Reggiani, 2011). Myofiber CSA was analyzed at four different ages: weaning (3 weeks), prepubertal (4 weeks), early adolescent (6 weeks) and young adult (12 weeks) (Fig. 1A,B). Consistent with previous reports, we found that myofiber CSA increases significantly post-weaning (Fig. 1C,D; Fig. S1) (Gokhin et al., 2008; White et al., 2010). However, the vast majority of this increase was readily apparent by early adolescence. Specifically, EDL and SOL CSA more than doubled between 3 and 6 weeks, from an average of 470 μm^2^ to 1247 μm^2^ and 767 μm^2^ to 1672 μm^2^, respectively (Fig. 1C,D; Fig. S1). In order to gain further insight into the dynamics of myofiber growth, CSA values were binned into 250 μm^2^ increments for frequency distribution analysis. This revealed the progressive appearance of larger myofibers during prepubertal growth, in both EDL and SOL muscles. At 3 weeks, 54% of EDL myofibers had a CSA between 250 and 500 μm^2^, and by 4 weeks this proportion declined substantially to 29% (Fig. 1E). Further refinement to 14% and 10% was observed in 6- and 12-week myofibers, respectively (Fig. 1E). At 6 weeks, fibers larger than 1250 μm^2^ were readily apparent, having been rarely observed at the 3- and 4-week time points. Additionally, the establishment of larger fibers was very similar to the distribution seen in the 12-week young adult. A similar trend was found in SOL muscles. At weaning, 52% of SOL myofibers had a CSA between 500 and 750 μm^2^ (Fig. 1F). With maturation, a robust drop to 31% was already observed at 4 weeks. By early adolescence, this proportion had declined to negligible levels, as was observed in young adult SOL myofibers (0-2%) (Fig. 1F). The appearance of fibers with a CSA greater than 1250 μm^2^ was established by 6 weeks of age, similar to the EDL. Therefore, in representative fast- and slow-contracting muscles, there is significant myofiber growth during prepuberty, with the establishment of adult myofiber sizes in early adolescence.

### Myonuclear content increases during prepubertal growth and is tightly correlated with myofiber size

**Fig. 2.**
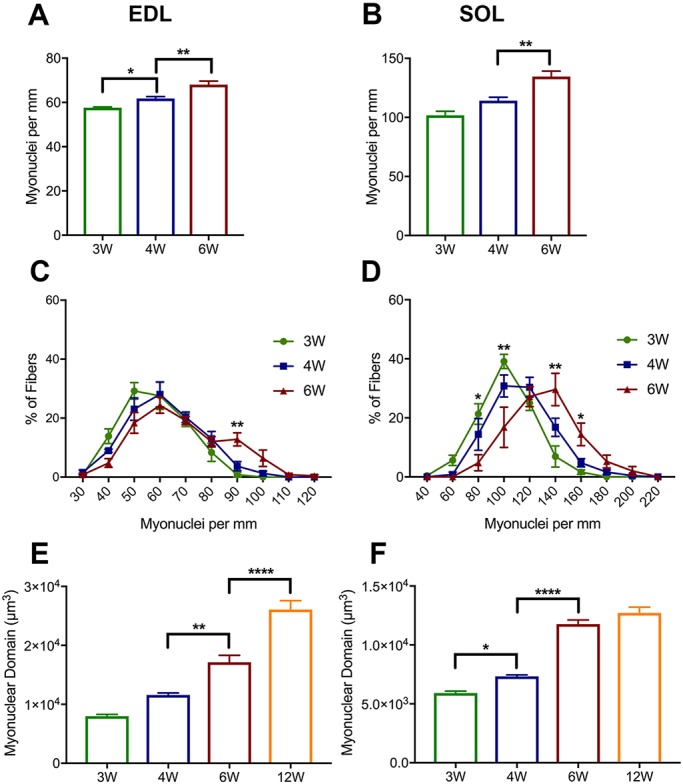
**Myonuclear number and domain increase during prepuberty**. (A,B) Bar graphs of myonuclear content in EDL (A) and SOL (B) at 3-, 4- and 6-week time points. *n*=5 mice per group. *P<0.05, **P<0.01 (one-way ANOVA with Tukey's test). (C,D) Frequency distribution of myonuclear content in EDL (C) and SOL (D) at 3-, 4- and 6-week time points. *n*=5 mice. Two-way ANOVA, Tukey. *P<0.05, **P<0.01 (significance between 4 and 6 weeks). Statistical comparisons are provided in Table** **S1. (E,F) Myonuclear domain in EDL (E) and SOL (F) at 3-, 4-, 6- and 12-week time points. *n*=5 mice per group. *P<0.05, **P<0.01, ****P<0.0001 (one-way ANOVA with Tukey's test).

Myofibers are multinucleated cells that contain hundreds of uniformly distributed myonuclei capable of transcriptional activity (Fig. 1A,B) (Horsley and Pavlath, 2004). It is well understood that SCs are the source of additional myonuclei during late embryonic and postnatal development (Moss and Leblond, 1970). Additionally, human studies suggest that myonuclear content increases with age during the first 18 years of life (Verdijk et al., 2014). In order to determine whether myonuclear number increases during murine prepubertal growth, we counted the number of myonuclei per millimeter (MN/mm) of individual EDL and SOL myofibers at weaning (3 weeks), prepuberty (4 weeks) and early adolescence (6 weeks). In the EDL, average myonuclear number increased significantly between weaning (58 MN/mm) and early adolescent ages (68 MN/mm) (Fig. 2A, Fig. S2A). Similarly, SOL myonuclear number increased from 102 MN/mm at weaning to 135 MN/mm at early adolescence (Fig. 2B, Fig. S2B). To better understand myonuclear number changes during prepuberty, we again used frequency distribution analysis (Fig. 2C,D). Myofibers were binned based on the number of myonuclei per millimeter. In both the EDL and SOL, the frequency distribution shifted rightward as time progressed from 3 to 6 postnatal weeks. At 3 weeks, the EDL had 29% of myofibers with 40-50 MN/mm, which decreased significantly to 18% at 6 weeks. At the 6-week time point there was an induction of fibers with greater than 80 MN/mm, which were not observed at 3 and 4 weeks (Fig. 2C). A similar, albeit more drastic, trend was apparent in the SOL. A rightward shift in SOL myonuclear content was observed from prepuberty to early adolescence, with an increase in the percentage of myofibers with greater than 120 MN/mm at 6 weeks of age (Fig. 2D).

Next, we interrogated myonuclear domain (MND), which is defined as the ratio of myofiber cytoplasm to myonuclear number (Allen et al., 1999). This parameter has been studied under numerous conditions, including development, aging, denervation, and exercise paradigms (Aravamudan et al., 2006; Bruusgaard et al., 2006; Damas et al., 2018; Mantilla et al., 2008; White et al., 2010). In both EDL and SOL, MND increased significantly throughout prepuberty. Between 4 and 6 weeks, the EDL and SOL MNDs increased by ∼1.5- and 1.6-fold, respectively (Fig. 2E,F). There was no significant difference in MND when comparing 6- and 12-week SOL myofibers. However, we observed a significant 1.5-fold increase between the 6- and 12-week EDL myofibers. Thus, myonuclear domain and number increase in both EDL and SOL myofibers during prepuberty.

**Fig. 3.**
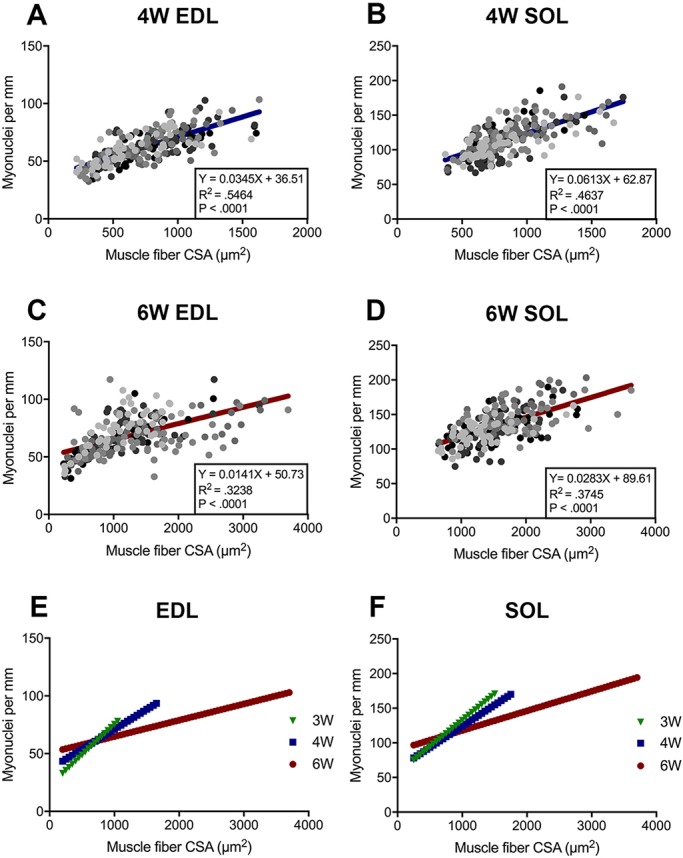
**Myonuclear content is correlated with larger fiber CSA during prepubertal growth**. (A,B) Linear regression analysis displaying correlation between CSA and myonuclear content at 4 weeks in EDL (A) and SOL (B). Each circle represents one myofiber. *n*=5 mice. EDL, 246 myofibers; SOL, 250 myofibers. (C,D) Linear regression analysis displaying correlation between CSA and myonuclear content at 6 weeks in EDL (C) and SOL (D). Each circle represents one myofiber. *n*=5 mice. EDL, 250 myofibers; SOL, 250 myofibers. (E,F) Overlay of 3-week, 4-week and 6-week slopes for EDL (E) and SOL (F).

We next investigated whether there is a correlation between myonuclear number and the CSA of myofibers during prepubertal growth (Fig. 3). Previous studies have looked at the relationship between myofiber CSA and myonuclear content during chicken skeletal muscle development, as well as mouse skeletal muscle aging and functional overload models (Brack et al., 2005; Egner et al., 2016; Moss, 1968). However, this analysis has not been carried out for mice during prepubertal or early adolescent ages. The CSA of 3-, 4- and 6-week EDL and SOL myofibers was plotted against their respective number of myonuclei per millimeter (Fig. 3, Fig. S3). At 4 weeks of age, there was a significant and tight correlation between myofiber size and myonuclear number in both EDL and SOL muscles (R^2^ values of 0.5464 and 0.4637, respectively). In the EDL, the myofibers with the largest CSA (∼1600 μm^2^) had on average 100 MN/mm, whereas the smallest (∼300 μm^2^) had on average 35 MN/mm (Fig. 3A). This trend was consistent in the SOL, with the largest myofibers (∼1750 μm^2^) having 175 MN/mm and smallest (∼400 μm^2^) having 70 MN/mm (Fig. 3B). Although there was a significant correlation between myonuclear number and CSA at 6 weeks, this association was not as tight as that observed at prepuberty (R^2^ of 0.3238 and 0.3745 for EDL and SOL, respectively) (Fig. 3C-F). This was more evident in the larger EDL myofibers, which had greater variation in myonuclear number. These data suggest that myonuclear accretion is an important cellular mechanism that contributes to prepubertal skeletal muscle hypertrophic growth.

### Transcriptome analysis reveals substantial changes in skeletal muscle gene expression during prepuberty

**Fig. 4.**
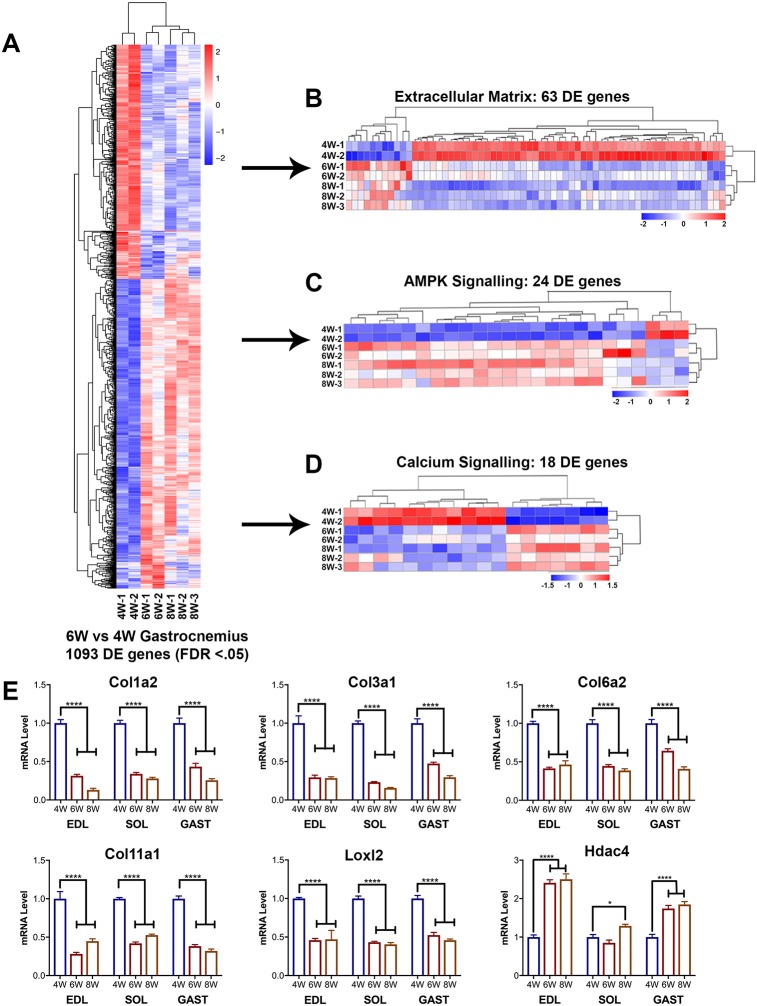
**RNAseq reveals substantial gene expression changes between 4 and 6 postnatal weeks.** (A) Heat map displaying significantly differentially expressed (DE) genes (FDR<0.05) from RNAseq of 4-, 6- and 8-week gastrocnemius muscles. *n*=2-3 mice per group. 1093 DE genes (6 weeks versus 4 weeks). Upregulated genes are shown in red and downregulated genes in blue. (B-D) Heat map displaying 6- versus 4-week DE genes related to the extracellular matrix (GO: 0044420) (B), AMPK signaling (IPA: AMPK-signaling pathway) (C) and calcium signaling (IPA: calcium-signaling pathway) (D). Individual genes are provided in Fig. S4A and Table S2. (E) RT-qPCR of RNAseq targets from 4-, 6- and 8-week EDL, SOL and gastrocnemius (GAST) muscles. mRNA level is shown relative to 4 weeks and normalized to *Gapdh*. *n*=3-5 mice per group. *P<0.05, ****P<0.0001 (one-way ANOVA with Bonferroni correction).

Because myofiber CSA and myonuclear content analysis revealed extensive growth during prepuberty, we next examined gene expression changes during this time. Genome-wide RNAseq was performed on 4-, 6- and 8-week-old C57BL/6 gastrocnemius muscles (Fig. 4). Initially comparing the 4- and 6-week time points, 1093 differentially expressed (DE) genes were identified (FDR<0.05) during this 2-week span of development (Fig. 4A). The 1093 DE genes were inputted into Ingenuity Pathway Analysis (IPA) for canonical pathway analysis. Among the top significantly changed pathways, integrin, calcium and AMPK signaling (z scores: −1.46, 1.897 and 2.065, respectively) are recognized pathways important for muscle function and development. Looking at integrin signaling more broadly, we examined ECM changes within the gene ontology term (GO: 0044420). In total, there were 63 DE ECM genes at 6 weeks, most of which were downregulated, compared with expression at 4 weeks (Fig. 4B, Fig. S4A). Genes involved in AMPK signaling, a master regulator of cell metabolism, were more highly expressed at 6 weeks of age (Fig. 4C, Fig. S4A). Calcium signaling, predicted to be activated by IPA, had a more uniform distribution of up- and downregulated genes at 6 weeks (Fig. 4D, Fig. S4A). However, ryanodine receptor 1 (*Ryr1*) was significantly upregulated at 6 weeks, which could account for the induction of calcium signaling predicted by IPA (Hernandez-Ochoa et al., 2015). Enriched gene ontology terms were analyzed and many of the biological components were related to development/skeletal muscle maturation (Fig. S4B). Additionally, cellular and molecular gene ontology terms were enriched in genes associated with collagen, the ECM, calcium signaling, and other processes related to skeletal muscle development (Fig. S4B).

To further understand gene expression changes between prepuberty and early adulthood, 4- and 8-week gastrocnemius RNA was compared. The results were similar to that of the 4- versus 6-week comparison but more pronounced. Between 4 and 8 weeks, we found 2258 DE genes (FDR<0.05). IPA indicated many similar enriched pathways: integrin, calcium, and AMPK signaling (z scores: −1.342, 3.024 and 3.000, respectively) were all within the top nine altered pathways (Fig. S5A). Interestingly, when 6- and 8-week gastrocnemius RNA was compared, the total number of DE genes (FDR<0.05) was reduced to only 163 (Fig. S5B). Thus, not only does extensive myofiber growth and myonuclear addition occur between 4 and 6 weeks (Figs 1-3), but our RNAseq analysis demonstrates that there are significant changes in muscle gene expression, consistent with robust and relatively rapid maturation during this period. This demonstrates that the transition from prepuberty to adolescence occurs through a critical switch point in terms of gene expression.

To validate the observed changes in gene expression, we performed RT-qPCR on relevant targets (*Col1a2*, *Col3a1*, *Col6a2*, *Col11a1*, *Hdac4*, *Loxl2*) in EDL, SOL and gastrocnemius muscles (Fig. S4A). Recent RNAseq analysis demonstrates these skeletal muscles differ in terms of gene expression, including genes related to contractile capability (Terry et al., 2018). Despite these differences, when *Col1a2*, *Col3a1*, *Col6a2*, *Col11a1*, *Hdac4* and *Loxl2* expression was examined, similar trends were observed across all three muscles when comparing the 6- and 8-week time points with 4 weeks (Fig. 4E).

### Prepubertal skeletal muscle growth is characterized by SC-derived myonuclear contribution that declines upon puberty onset

**Fig. 5.**
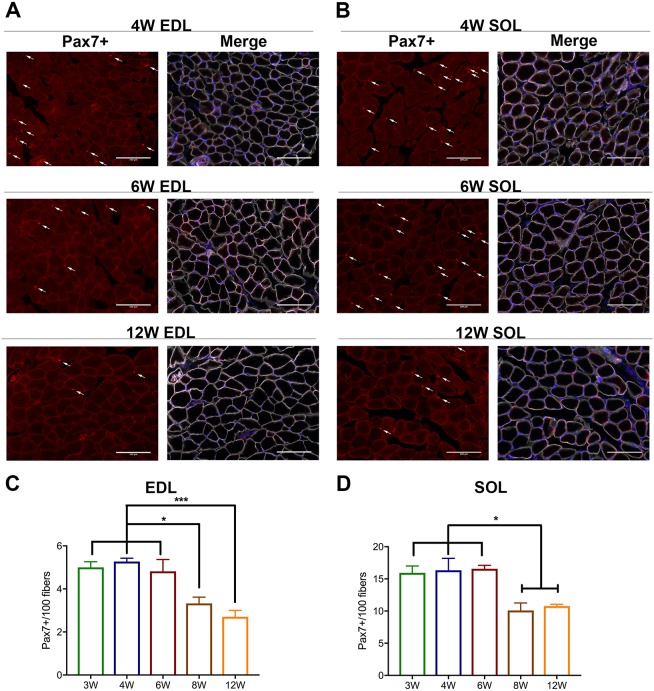
**Examination of SC pool size between prepuberty and young adulthood.** (A,B) Representative cross-sections of 4-, 6- and 12-week EDL (A) and SOL (B) muscles stained with Pax7 (red) and laminin (white) antibodies and DAPI (blue). Arrows indicate SCs. Scale bars: 100 μm. (C,D) Quantification of Pax7^+^ SC number (per 100 fibers) in 3-, 4-, 6-, 8- and 12-week EDL (C) and SOL (D) muscles. *n*=3-5 mice per group. Three sections, with three to six fields of view (20×), were averaged per mouse. *P<0.05, ***P<0.001 (one-way ANOVA with Tukey's test).

To determine whether myonuclear accretion and gene expression changes between 4 and 6 weeks were accompanied by modifications in SC pool size, we counted the number of Pax7-expressing SCs (per 100 fibers) in 3-, 4-, 6-, 8- and 12-week EDL and SOL cross-sections (Fig. 5A,B). There was no difference in SC number between 3 and 6 weeks of age (Fig. 5C,D). Therefore, myonuclear accretion and modifications in gene expression between 4 and 6 weeks were not accompanied by significant alterations in SC pool size. At 8 weeks, a significant decrease in SC number was observed in EDL and SOL (33% and 37% reduction, respectively). There was no significant difference when comparing the 8- and 12-week time points indicating that adult SC pool size is established at 8 weeks (late adolescence/young adulthood) (Dutta and Sengupta, 2016; Verdijk et al., 2014).

**Fig. 6.**
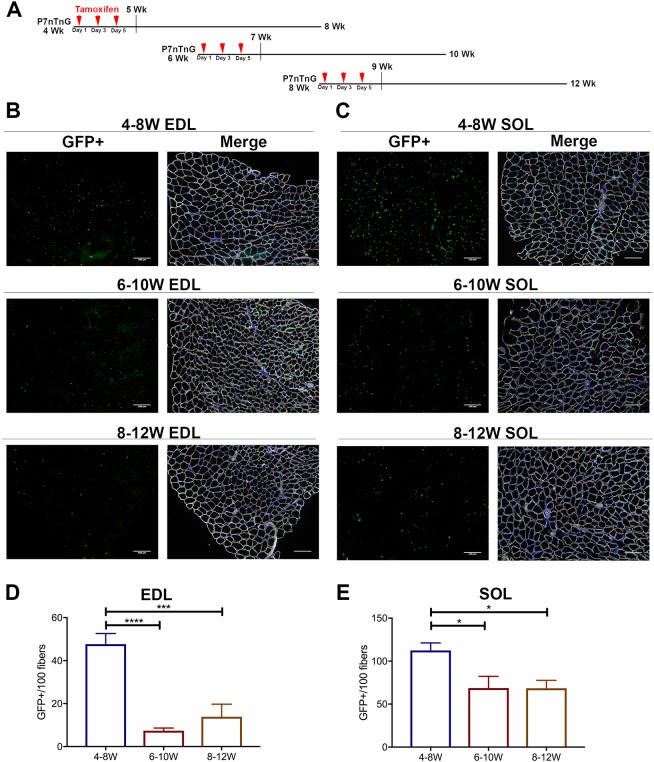
**SCs contribute to EDL and SOL muscles during prepubertal growth.** (A) Scheme representing tamoxifen administration at 4, 6 or 8 weeks with tissue harvest at 8, 10 or 12 weeks, respectively. (B,C) Representative cross-sections of 4-8, 6-10 and 8-12 week EDL (B) and SOL (C) muscles following tamoxifen injection (at 4, 6 or 8 weeks) to label SCs and derived myonuclei. Sectioned are stained with GFP (green), DAPI (blue) and laminin antibody (white). Scale bars: 100 μm. (D,E) Quantification of GFP^+^ myonuclei (per 100 fibers) in 4-8, 6-10 and 8-12 week EDL (D) and SOL (E) cross-sections. *n*=3-8 mice per group. *P<0.05, ***P<0.001, ****P<0.0001 (one-way ANOVA with Tukey's test).

To examine whether Pax7-expressing SCs are a source of prepubertal myonuclei, we utilized a previously characterized *Pax7^CreERT2/+^*; *Rosa26^nTnG/+^* (P7nTnG) mouse (Liu et al., 2017; Prigge et al., 2013). The P7nTnG mouse ubiquitously expresses a loxP-flanked nuclear Td-tomato fluorescent red reporter. Upon tamoxifen injection, the nuclear Td-tomato reporter is excised to indelibly label Pax7^+^ SCs and their derived cells with nuclear GFP (nGFP). To initially label SCs and track derived progenitor fate, P7nTnG mice were given tamoxifen at prepuberty (4 weeks), early adolescence (6 weeks) or young adulthood (8 weeks) and sacrificed 4 weeks thereafter (Fig. 6A). Upon tamoxifen administration at 4 weeks and examination of skeletal muscles at 8 weeks, we observed substantial SC-derived nGFP^+^ myonuclear contribution in both EDL and SOL (∼50 and 110 nGFP^+^/100 fibers, respectively) (Fig. 6B-E). As we only found approximately three and ten SCs/100 fibers in 8-week-old EDL and SOL sections, respectively (Fig. 5C,D), an overwhelming proportion of nGFP^+^ cells were indeed SC-derived myonuclei. The administration of tamoxifen at 6 and 8 weeks revealed a marked decline in SC-derived nGFP^+^ myonuclear contribution (Fig. 6B-E). Similarly, other lower limb, upper limb and trunk skeletal muscles, such as the tibialis anterior, plantaris, gastrocnemius, quadriceps and diaphragm, all exhibited extensive SC-derived nGFP^+^ myonuclear contribution upon tamoxifen administration at 4 compared with 6 weeks of age (Fig. S6). These data demonstrate that puberty onset is a seminal event in ceasing the contribution of SC-derived myonuclei during postnatal growth (Kim et al., 2016). Furthermore, we demonstrate that SCs are the principal source of myonuclear accretion associated with increased myofiber CSA during prepubertal myofiber hypertrophic growth.

### Prepubertal SC depletion impedes myofiber hypertrophic growth and reduces myonuclear number

**Fig. 7.**
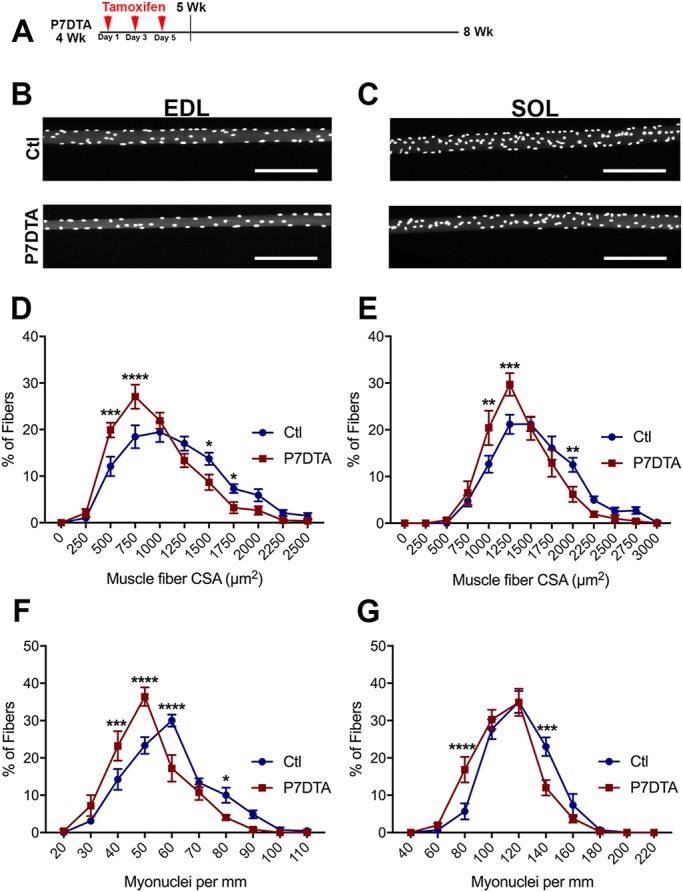
**Prepubertal SC ablation leads to similar declines in myofiber hypertrophic growth and myonuclear number.** (A) Illustration of P7DTA scheme: tamoxifen was administered at 4 weeks and tissue harvested at 8 weeks. (B,C) Representative images of control (Ctl) and P7DTA EDL (B) and SOL (C) myofibers. Scale bars: 200 μm. (D,E) Frequency distribution of myofiber CSA of Ctl and P7DTA EDL (D) and SOL (E) myofibers. *n*=9-10 mice per group for EDL and 6-7 per group for SOL. *P<0.05, **P<0.01, ***P<0.001, ****P<0.0001 (two-way ANOVA with Fisher's LSD test). (F,G) Frequency distribution of myonuclear number (MN/mm) of Ctl and P7DTA EDL (F) and SOL (G) myofibers. *n*=7 mice per group for EDL and 6-7 per group for SOL. *P<0.05, ***P<0.001, ****P<0.0001 (two-way ANOVA with Fisher's LSD test).

Next, we wanted to ascertain the functional relevance of SC-derived myonuclear accretion to prepubertal myofiber growth. For this purpose, we utilized a *Pax7^CreER/+^*; *Rosa26^DTA/+^* (P7DTA) mouse (Keefe et al., 2015; Liu et al., 2017, 2015; Murphy et al., 2011; Wu et al., 2006). This mouse line enables expression of diphtheria toxin A (DTA) in SCs upon tamoxifen injection, causing SC depletion. Tamoxifen was injected three times (every other day) beginning at 4 weeks and mice were sacrificed at 8 weeks of age (Fig. 7A). This strategy led to efficient SC depletion based on quantification of Pax7-expressing cells in P7DTA EDL and SOL muscles (Fig. S7A-C).

Using the P7DTA model, we first assayed myofiber CSA and myonuclear number for EDL and SOL muscles (Fig. 7B,C). Using frequency analysis, we observed a significant induction of myofibers with a smaller CSA, between 250 and 750 μm^2^, in the P7DTA EDL (Fig. 7D). Compared with control, there was a leftward shift in the distribution of P7DTA EDL myofiber CSA (Fig. 7D). In addition to having smaller myofibers, we observed that the P7DTA EDL myofibers had fewer myonuclei than their control counterparts (Fig. 7F). The P7DTA EDL myofibers had approximately 75% of myofibers with fewer than 50 MN/mm. Furthermore, we observed a leftward shift in the distribution of the P7DTA EDL MN/mm curve in comparison with control. On average, P7DTA EDL myofiber CSA and myonuclear number were reduced, by 19% and 15%, respectively, compared with control (Fig. S8A,B). Similar trends were observed in the P7DTA SOL muscle. The P7DTA SOL had significantly higher proportions of myofibers with a relatively smaller CSA (Fig. 7E). In addition, the P7DTA SOL had higher proportions of myofibers with relatively fewer myonuclei (Fig. 7G). On average, the P7DTA SOL myofibers had smaller CSAs and fewer myonuclei, by 15% and 10%, respectively, compared with control (Fig. S8C,D). Therefore, targeted prepubertal depletion of Pax7-expressing SCs leads to a reduction of EDL and SOL myofiber CSA that is associated with a similar loss of myonuclei.

### Prepubertal SC depletion causes force generation deficits in fast- and slow-contracting muscles

**Fig. 8.**
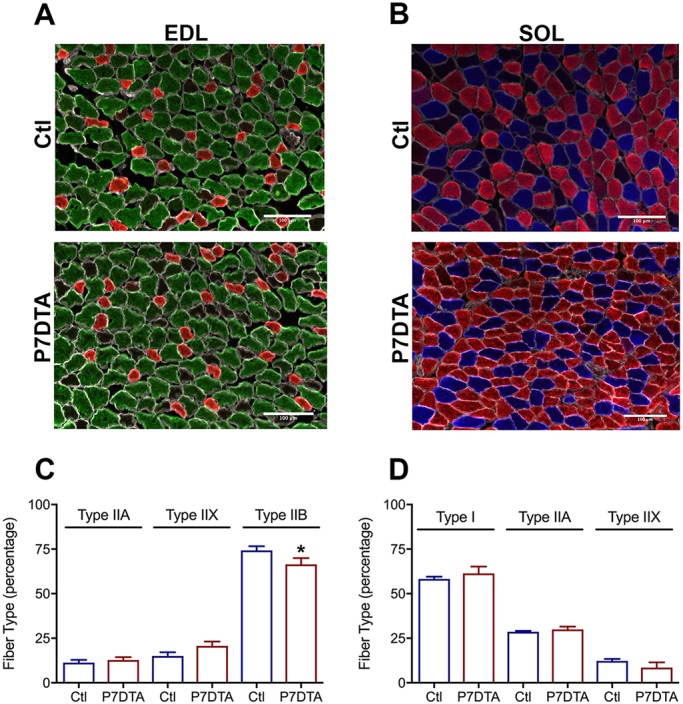
**Minimal fiber-type changes with prepubertal SC ablation.** (A,B) Representative images of control (Ctl) and P7DTA EDL (A) and SOL (B) fiber-type immunostaining. MYHC type IIB fibers, green; IIX, black; IIA, red; I, blue; laminin, white. Scale bars: 100 μm. (C,D) Quantification of fiber-type percentages for Ctl and P7DTA EDL (C) and SOL (D). *n*=5-7 mice per group for EDL and 3-4 per group for SOL. *P<0.05 (one-way ANOVA with Fisher's LSD test).

Most skeletal muscles possess a mix of distinct myofiber types identified by the expression of individual myosin heavy chains: type I, IIA, IIX and IIB (Schiaffino and Reggiani, 2011). Skeletal muscle myosin heavy chains regulate the speed of myofiber contraction. Along a continuum, type I fibers contract more slowly and generate less force compared with type IIB myofibers (Mantilla and Sieck, 2003). Typically, a large reduction of type IIB myofibers is observed upon consequential mouse neuromuscular disruption, such as denervation or neuromuscular disease, which can be exacerbated by SC depletion (Chakkalakal et al., 2012b; Frey et al., 2000; Liu et al., 2015). Therefore, we investigated whether prepubertal SC depletion had any effect on fiber type in EDL and SOL muscles. Although no extreme alterations in myofiber type were observed upon SC depletion at 4 weeks, a modest (5%), albeit significant, reduction in type IIB myofibers was found in EDL muscles (Fig. 8A,C). A non-significant trend for elevated proportions of type IIA and IIX myofibers accompanied this reduction. Prepubertal SC depletion had minimal effects on SOL fiber-type distribution (Fig. 8B,D). Consistent with no major alterations in myofiber type, analysis of neuromuscular junction (NMJ) innervation integrity did not reveal any observable neuromuscular disruption (Fig. S9).

**Fig. 9.**
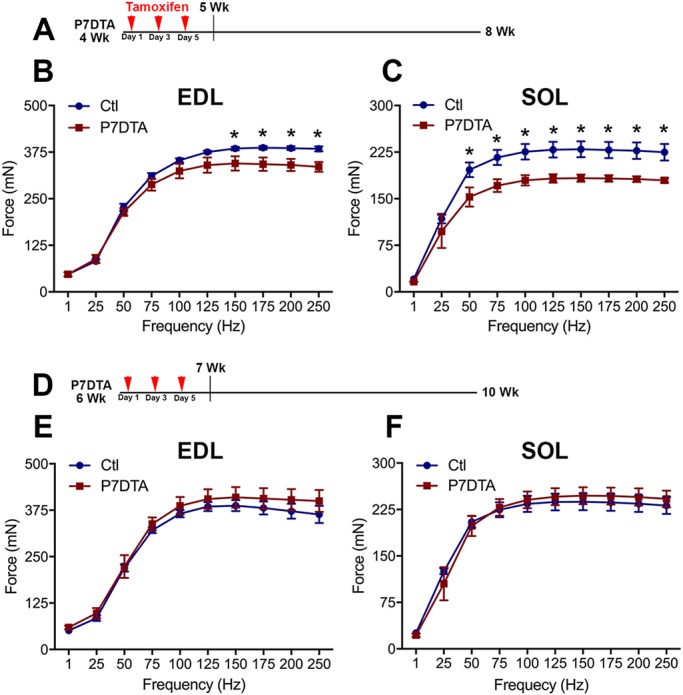
**Force generation deficits following prepubertal SC ablation.** (A) Illustration of 4W-P7DTA scheme: tamoxifen was injected at 4 weeks and tissue harvested at 8 weeks. (B,C) Absolute force values generated by EDL (B) and SOL (C) control (Ctl) and 4W-P7DTA mice. *n*=3-4 mice per group. *P<0.05 (Fisher's LSD test). (D) Illustration of 6W-P7DTA scheme: tamoxifen was injected at 6 weeks and tissue harvested at 10 weeks. (E,F) Absolute force values generated by EDL (E) and SOL (F) Ctl and 6W-P7DTA mice. *n*=5-7 mice per group.

Because prepubertal SC depletion led to impaired myofiber hypertrophic growth and loss of myonuclei, we next sought to determine whether these phenotypes were associated with intrinsic declines in skeletal muscle function. For this purpose, we employed an *ex vivo* skeletal muscle contractile system to assay EDL and SOL muscle intrinsic force generation capacity and contractile properties (Liu et al., 2017, 2015). In comparison with control muscles, we observed significant deficits in absolute force generation in 8-week-old EDL and SOL muscles that had been depleted of SCs during prepuberty (4 weeks) (Fig. 9A-C, Fig. S10A). In contrast, tamoxifen-induced SC depletion during early adolescence (6 weeks) did not reveal deficits in force generation in P7DTA EDL or SOL muscles (Fig. 9D-F). Examination of time-to-peak tension, an indicator of contractile speed, did not reveal any significant differences between P7DTA EDL or SOL muscles compared with controls (Fig. S10B). Therefore, the modest myofiber-type transitions observed in the P7DTA EDL were not sufficient to alter contractile speed (Fig. 8C). Another parameter that can influence intrinsic skeletal muscle force generation is myofiber number. We observed no difference in myofiber number between control and P7DTA EDL and SOL muscles depleted of SCs during prepuberty (Fig. S7D). Collectively, these data demonstrate that prepubertal SC depletion leads to functional deficits primarily associated with loss of myonuclei and reduction in myofiber CSA.

As a loading force, skeletal muscle can influence bone development and healing (Brotto and Bonewald, 2015; Harry et al., 2008; Pedersen, 2011). This relationship appears to be even more pivotal during postnatal growth, as increases in muscle force correlate with bone strength (Fricke and Schoenau, 2007; Rauch et al., 2004; Schoenau and Frost, 2002). Therefore, we examined whether bone biomechanical and intrinsic properties are altered in response to skeletal muscle force deficits, upon prepubertal SC ablation. We found no difference in tibial cortical or trabecular thickness or bone volume in P7DTA compared with control mice (Fig. S11A-D). Also, there was no difference in tibial length between P7DTA and control (data not shown). However, biomechanical testing revealed that there is a trend for P7DTA tibias to score lower in terms of bone toughness and strength (Fig. S11E,F).

## DISCUSSION

Lineage-tracing and depletion studies have identified Pax7-expressing SCs as the principal source of myonuclei during early development (Hutcheson et al., 2009; Moss and Leblond, 1970; Relaix and Zammit, 2012). Previous studies have reported mitotic activity and increases in myonuclear number up to sexual maturity/young adulthood in mouse and rat models (Cardasis and Cooper, 1975; Enesco and Puddy, 1964; Kelly, 1978; Williams and Goldspink, 1971). However, the functional relevance of SC contributions from prepuberty to early adolescence had not been examined. In this study, we observed significant increases of mouse myofiber size and myonuclear number between prepuberty and early adolescence/puberty onset (4-6 weeks of age). This time point proved to be crucial to muscle development, with significant changes in muscle ECM, calcium handling and AMPK signaling. Lastly, SC contribution to myofibers during prepuberty and early adolescence was essential, with SC depletion resulting in reduced hypertrophic growth, loss of myonuclei, and force deficits.

Murine muscle development was examined between 3 and 12 weeks of age. During this time, myofiber CSA increased significantly in both fast-contracting EDL and slow-contracting SOL muscles, particularly between 4 and 6 weeks (Fig. 1, Fig. S1). Additionally, we found myofibers to transition in size throughout development, with the establishment of a heterogeneous adult-like population at 6 weeks of age. The hypertrophy we observed between prepuberty and early adolescence (4 and 6 weeks) was associated with significant myonuclear accretion, expansion of MND, and correlated with myonuclear number (Figs 2 and 3; Figs S2 and S3). Other studies have reported results indicative of increases in myonuclear number during similar stages of growth (Cardasis and Cooper, 1975; Neal et al., 2012). White et al. (2010), found myonuclear accretion to stagnate at weaning in mouse EDL myofibers. In contrast, we found increases in EDL and SOL myonuclear number from weaning to early adolescence. Considering the counts conducted in this study were normalized per unit length and myofiber length does increase into adulthood, our data may underestimate the scope of myonuclear accretion. Indeed, a recent report demonstrated a sizable increase in absolute myonuclear number when comparing 3- and 5-week-old EDL myofibers (Huh et al., 2017). However, consistent with White et al., we observed expansion of MND during this period of growth. Collectively, based on the extent of myofiber CSA, MND and myonuclear number changes, other mechanisms in addition to SC-derived myonuclear accretion are likely to be effectors of prepubertal myofiber hypertrophic growth.

Utilizing genome-wide RNAseq, we found there to be significant changes in the skeletal muscle transcriptome during prepuberty and adolescence (4-8 weeks of age). With 1093 DE genes (FDR<0.05), a multitude of changes related to skeletal muscle development had occurred between 4 and 6 weeks of age. The most significant of these were related to calcium handling, AMPK signaling and ECM composition (Fig. 4, Figs S4 and S5). Recently, Brinegar et al. (2017) investigated the changes in gene expression and splicing during postnatal development, specifically related to calcium handling; utilizing RNAseq, this group found 1496 DE genes (±2.0 fold change) between 4 and 22 weeks. We observed a similar trend; however, the vast majority of changes occurred between 4 and 8 weeks, emphasizing the importance of this short, but crucial, period of skeletal muscle growth. AMPK signaling was also predicted to be increased at both 8 and 6 weeks, compared with the prepubertal 4-week time point. AMPK signaling is a well-defined regulator of cell metabolism, playing a pivotal role in skeletal muscle energy homeostasis (Hardie and Sakamoto, 2006; Kjobsted et al., 2018). Additionally, AMPK is known to directly affect SC fate decisions (Mounier et al., 2013; Theret et al., 2017). It is likely that during this time of rapid growth and development, AMPK activation is necessary to facilitate changes in skeletal muscle energy demands.

Our RNAseq analysis also predicted significant ECM remodeling between 4 and 6 weeks of age. We found 63 of the genes within the extracellular matrix category (GO: 0044420) to be differentially expressed, a vast majority of them being downregulated at 6 weeks. The ECM has been shown to play an important role in the SC niche, regulating self-renewal and fate decisions (Thomas et al., 2015; Urciuolo et al., 2013). Urciuolo et al. (2013) investigated the role of collagen VI in the SC niche and found *Col6a1*^−/−^ mice to have deficits in SC self-renewal, both *in vitro* and *in vivo*; consistent with this study and decreased SC activity, *Col6a1* was significantly downregulated at early adolescence/puberty onset. Overall, it appears that remodeling of the ECM during prepuberty coincides with changes in SC activity. Further study is warranted to determine how prepubertal modulations in ECM composition can alter SC fate decisions and skeletal muscle maturation.

Enumeration of Pax7-expressing SC number revealed no changes between prepuberty and early adolescence (Fig. 5C,D). Unexpectedly, we found a significant decrease in EDL and SOL SC number at 8 weeks, late adolescence/young adulthood. This is consistent with robust SC activity up to puberty onset and the establishment of adult SC pool size at late adolescence. In support, utilizing a Pax7nTnG reporter mouse, we found a significant contribution of SC-derived nGFP^+^ myonuclei to skeletal muscle up to puberty onset, with considerable drop-off thereafter (Fig. 6, Fig. S6). In comparison with fast-contracting EDL muscles, we observed a more prolonged contribution of SC-derived myonuclei to slow-contracting SOL muscles up to adulthood (12 weeks of age). Pawlikowsi et al. (2015) reported similar results using a slightly different strategy to indelibly label SCs. Upon inducible labeling of SCs at 4 weeks and examination of SC fate 2 weeks thereafter, this group found SC contribution based on the incorporation of a cytoplasmic reporter in myofibers. Furthermore, Pawlikowski et al. observed that the EDL muscle reached a steady state of SC fusion at a much earlier age (8 weeks) in comparison with SOL muscles (12 weeks). Whether this distinction is due to the intrinsic nature of the SCs or to the myofibers requires further study.

Our results indicate the onset of puberty as the pivotal point in the reduction of SC contribution to developing myofibers. Kim et al. (2016) demonstrated that the induction of sex hormones (both androgens and estrogens) can induce Notch signaling in juvenile cycling SCs, converting SCs to their adult quiescent state. Additionally, interfering with the induction of sex hormones (either surgically or pharmacologically) at 2 weeks of age resulted in prolonged cycling and delayed entry into quiescence. Notch signaling has proven to be crucial for maintaining SC quiescence throughout adulthood. Postnatal SC-specific disruption of the Notch effector RBP-Jκ leads to loss of SCs owing to premature terminal differentiation (Bjornson et al., 2012; Mourikis et al., 2012). Therefore, a decrease in the contribution of SC-derived progenitors to skeletal muscle growth is likely to be linked to the induction of sex hormones and regulation of Notch signaling. Indeed, castration of adult mice leads to disruption of SC quiescence and an increased requirement for SC-derived progenitor-mediated skeletal muscle maintenance (Klose et al., 2018).

SC depletion studies have demonstrated the indispensable nature of SCs in muscle regeneration (Lepper et al., 2011; Murphy et al., 2011; Relaix and Zammit, 2012). Additionally, SC ablation has been shown to prevent myofiber hypertrophy in models of functional overload and to promote denervation and age-related myofiber atrophy, in some but not all muscles (Egner et al., 2016; Fry et al., 2015; Keefe et al., 2015; Liu et al., 2017, 2015; McCarthy et al., 2011). However, there was yet to be a study targeting SCs during prepuberty or early adolescence (3-6 weeks). We found significant deficits in myofiber size and myonuclear content in both EDL and SOL muscles 4 weeks after prepubertal SC depletion (Fig. 7, Fig. S8). The extent of these declines was similar. Therefore, the effects of prepubertal SC depletion on myofiber size were likely to be a consequence of myonuclear loss. In contrast, SC depletion well into adulthood leads to EDL myofiber atrophy and loss of myonuclei over a protracted period of time, whereas other muscles, such as the SOL, demonstrate modest, if any, phenotypes (Fry et al., 2015; Keefe et al., 2015; Liu et al., 2017). Although technical variations, including the timing and extent of SC depletion and myofiber examination methods, may explain some of these discrepancies, prepubertal SC depletion clearly has a more immediate impact on the morphology and function of distinct skeletal muscles.

In addition to deficits in myofiber CSA and myonuclear number, prepubertal SC depletion led to a reduction in intrinsic skeletal muscle force generation (Fig. 9, Fig. S10). During postnatal growth, increases in skeletal muscle force are associated with increased bone strength (Fricke and Schoenau, 2007; Rauch et al., 2004; Schoenau and Frost, 2002). We observed a non-significant trend toward decreased bone strength upon prepubertal SC depletion (Fig. S11). One possibility explaining the minimal decreases in bone strength could be that declines in skeletal muscle force are simply not severe enough. Another possibility is that reductions in bone strength, due to loss of myofiber size and myonuclei, may take a longer time to manifest.

Although prepubertal SC depletion impaired myofiber hypertrophic growth, myonuclear number and skeletal muscle function, this was not associated with any obvious disruption in NMJ integrity (Fig. S9). Similar to SC depletion in aging and androgen-deprived skeletal muscles, NMJ disruptions upon prepubertal SC depletion may require a longer period of time to appear (Klose et al., 2018; Liu et al., 2017). Alternatively, the timing of SC depletion may be a factor. Previous work has shown that the mid portions of myofibers demonstrate rudimentary forms of endplate specialization during embryonic and early postnatal growth, even when deprived of neural inputs (Lin et al., 2001; Liu and Chakkalakal, 2018). These data suggest that embryonic and early postnatal myofibers may have intrinsic programs for rudimentary NMJ formation. Immature myofibers have been observed to grow out from the center, possibly by the addition of myonuclei to their ends (Wang et al., 2010; Zhang and McLennan, 1995). Therefore, SC depletion that targets the addition of myonuclei at earlier stages may lead to more immediate NMJ disruptions at more primitive myofiber sites.

Heterogeneity exists among the SC population, with the potential for long-term renewal based on *Pax7* and *Myf5* expression (Kuang et al., 2007; Rocheteau et al., 2012). Additionally, all SCs do not contribute equally during postnatal growth, as label-retaining populations contribute less vigorously (Chakkalakal et al., 2014; Schultz, 1996). It will be important to identify more accurately these subpopulations and assess their role during prepubertal growth. It is possible that certain populations are more susceptible to insults during this time of development (Mozdiak et al., 1997). Our findings uncover the importance of progenitor-based skeletal muscle growth and maturation during prepuberty. The potential to mimic the raw, untamed power of juvenile SCs could prove to have important therapeutic benefits in both disease and aging.

## MATERIALS AND METHODS

### Animals

Wild-type C57BL/6J, *Pax7^CreERT2^* (017763), *Rosa26^nTnG^* (023035) and *Rosa26^DTA^* (009669) mice were obtained from Jackson Laboratories. *Rosa26^nTnG^* and *Rosa26^DTA^* were crossed with *Pax7^CreERT2^* mice to generate *Pax7^CreER/+^*; *Rosa26^nTnG/+^* (P7nTnG) (Liu et al., 2017; Prigge et al., 2013), *Pax7^CreER/+^*; *Rosa26^DTA/+^* (P7DTA) (Klose et al., 2018; Liu et al., 2017, 2015; Murphy et al., 2011), and control CreER-negative (Ctl) littermates. All mice used in this study were male. Tamoxifen (Sigma-Aldrich, T5648) was administered via intraperitoneal injection at a dose of 2.0 mg/day three times (every other day) to induce Cre recombination during juvenile and adolescent ages (4, 6 and 8 weeks). All animal procedures were conducted in accordance with institutional guidelines approved by the University Committee on Animal Resources, University of Rochester Medical Center.

### Antibodies

The following antibodies were used: Pax7 [mouse IgG1, 1:100, Developmental Studies Hybridoma Bank (DSHB)], laminin (rabbit, 1:1500, Sigma-Aldrich, L9393; rat, 1:1500, Sigma-Aldrich, L0663), GFP (rabbit, 1:400, Millipore AB3030P), BA-D5 (MyHC-I, mouse IgG2b, 1:40, DSHB), SC-71 (MyHC-IIA, mouse IgG1, 1:40, DSHB), BF-F3 (MyHC-IIB, mouse IgM, 1:40, DSHB), SV2 (synaptic vesicle protein-2, mouse IgG1, 1:100, DSHB), Znp-1 (synaptotagmin-2, mouse IgG2a, 1:200, DSHB), 2H3 (neurofilament, mouse IgG1, 1:200, DSHB), Alexa Fluor 488-conjugated α-bungarotoxin (1:1000, Thermo Fisher Scientific, B-13422), Alexa Fluor 405-conjugated goat anti-mouse IgG2b (1:1500, Thermo Fisher Scientific, A-21141), Alexa Fluor 488-conjugated goat anti-mouse IgM (1:1500, Thermo Fisher Scientific, A-21042), Alexa Fluor 594-conjugated goat anti-mouse IgG1 (1:1500, Thermo Fisher Scientific, A-21125), DAPI (1:3000), Alexa Fluor 488-conjugated goat anti-rabbit IgG (1:1500, Thermo Fisher Scientific, A-32731).

### Immunofluorescence (IF)

Muscles were dissected and incubated in 30% sucrose overnight at 4°C. Muscles were embedded in OCT (Tissue Tek) and flash frozen using dry-ice-cooled isopentane. Muscles were cryosectioned at 10 or 30 μm, for transverse or longitudinal sections, respectively. Muscle sections were fixed in 4% paraformaldehyde (PFA) for 3 min, if necessary (no PFA fixation for MyHC antibodies). Sections were permeabilized with PBS-T (0.2% Triton X-100 in PBS) for 10 min, blocked in 10% normal goat serum (NGS, Jackson ImmunoResearch) for 30 min at room temperature, and primary antibodies applied. If a mouse primary antibody was used, sections were blocked in 3% AffiniPure Fab fragment goat anti-mouse IgG (H+L) (Jackson ImmunoResearch) with 2% NGS in PBS at room temperature for 1 h. Primary antibody incubation in 2% NGS/PBS was performed at 4°C overnight. Secondary antibody incubation in 2% NGS/PBS was carried out at room temperature for 1 h. DAPI staining was utilized to label myonuclei. All slides were mounted with Fluoromount-G (SouthernBiotech). Sections were imaged using a Zeiss Axio Observer A.1 microscope (Liu et al., 2017, 2015; Paris et al., 2016).

### Fixed single fiber analysis

For single myofiber size and myonuclear analysis, whole limbs were fixed in 4% PFA for 48 h prior to muscle dissection. Fixed muscles were incubated in 40% NaOH for 2 h to induce dissociation (Brack et al., 2005; Liu et al., 2017). Single myofibers were gently titrated and washed in PBS prior to staining with DAPI.

### RNA isolation and RT-qPCR

Muscles were flash frozen in Trizol (Ambion) at dissection. Tissue was then homogenized using a Bullet Blender Gold (Nextadvance BB24-AU). RNA was isolated using an RNeasy Plus Mini Kit (Qiagen) following manufacturer's instructions. cDNA was synthesized using 500 or 1000 ng of RNA (from EDL/SOL or gastrocnemius, respectively) using qScript cDNA SuperMix (QuantaBio). RT-qPCR was performed on a Step One Plus Real Time PCR machine (Applied Biosystems) using PerfeCTa SYBR Green FastMix (QuantaBio). All reactions utilized the following thermal cycler conditions: 50°C for 2 min, 95°C for 2 min, 40 cycles of a two-step reaction, denaturation at 95°C for 15 s, and annealing at 60°C for 30 s. Experiments were standardized to *Gapdh*. Primers are provided in Table S3.

### RNAseq library construction

Total RNA concentration was determined with the NanoDrop 1000 spectrophotometer (NanoDrop) and RNA quality was assessed with the Agilent Bioanalyzer. Illumina compatible library construction was performed using the TruSeq Total Stranded RNA Sample Preparation Kit (Illumina) following manufacturer's protocols. Briefly, rRNA was depleted from 200 ng total RNA with RiboZero magnetic beads. Residual RNA, depleted of rRNA, was chemically fragmented following manufacturer's recommendation. First-strand cDNA synthesis was performed using random hexamer priming followed by second-strand cDNA synthesis using dUTP. End repair and 3′ adenylation was performed on the double-stranded cDNA. Illumina adaptors were ligated to both ends of the cDNA, purified by gel electrophoresis and amplified with PCR primers specific to the adaptor sequences to generate amplicons of approximately 200-500 bp in size. The amplified libraries were hybridized to the Illumina single-end flow cell and amplified using the cBot (Illumina). Approximately, 40 million single-end reads of 100 nt were generated for each sample.

### NGS data processing and alignment

Raw reads generated from the Illumina HiSeq2500 sequencer were demultiplexed using bcl2fastq version 2.19.0. Quality filtering and adapter removal are performed using Trimmomatic version 0.36 with the following parameters: ‘TRAILING:13 LEADING:13 ILLUMINACLIP:adapters.fasta:2:30:10 SLIDINGWINDOW:4:20 MINLEN:15’ Processed/cleaned reads were then mapped to the *Mus musculus* reference sequence (GRCm38.p5) with STAR_2.5.2b with the following parameters: ‘—twopassMode Basic —runMode alignReads —genomeDir ${GENOME} —readFilesIn ${SAMPLE} —outSAMtype BAM SortedByCoordinate —outSAMstrandField intronMotif —outFilterIntronMotifs RemoveNoncanonical’. Read counts per gene were derived in a strand-specific manner using featureCounts from the subread-1.5.03 package with the following parameters: ‘-s 2–t exon –g gene_name’. Differential expression analysis and data normalization was performed using DESeq2-1.14.1 with an adjusted *P*-value threshold of 0.05 within an R v3.3.2 environment.

### Quantification of myofiber-type composition

Myofiber-type percentage of EDL and SOL was determined using an ImageJ plug-in (Liu et al., 2017). The plug-in is available at https://github.com/aidistan/ij-fibersize.

### *Ex vivo* muscle force generation assay

Muscle force generation capacity was analyzed in EDL and SOL muscles using an ASI muscle contraction system (Aurora Scientific) (Klose et al., 2018; Liu et al., 2017, 2015; Wei-Lapierre et al., 2013). Mice were anesthetized by ketamine (88 mg/kg) and xylazine (8 mg/kg) injection, and the TA muscle was removed. EDL and SOL were dissected, adjusted to optimal length (L_o_), and tested at different frequencies to determine absolute force values. Muscle force was recorded and analyzed using Dynamic Muscle Control and GraphPad Prism software. Cross-sectional area was calculated using the equation of cross-sectional area=muscle mass/[muscle density (1.06 g/cm^3^)×optimal fiber length (0.44×L_o_)] (Hakim et al., 2011).

### Confocal IF microscopy and NMJ analysis

Longitudinal sections were sectioned at 30 μm and stained with fluorescently conjugated α-bungarotoxin and antibodies for synaptic vesicle protein 2 (SV2), synaptotagmin-2 (Znp-1) and neurofilament (2H3) to label acetylcholine receptor and nerve terminals, respectively (Liu et al., 2017, 2015). Sections were observed on an Olympus Fluoview 1000 confocal microscope with 60× objective at a 1.0-μm step-size to determine innervation status.

### Bone microCT and biomechanical testing

Following sacrifice, the tibia was isolated and cleaned of excess soft tissue. Tibias were stored at 4°C in PBS overnight, prior to micro-computed tomography (microCT) and biomechanical testing. For microCT, tibias were imaged using a VivaCT 40 tomograph (Scanco Medical). Scanco analysis software was utilized for volume quantification. Trabecular bone versus total volume (BV/TV), cortical bone versus total volume (BV/TV), trabecular thickness and cortical thickness, were determined for tibias. For biomechanical testing, tibias were subjected to torsional testing. The ends of the tibias were cemented (Bosworth Company) in aluminium tube holders and tested using an EnduraTec TestBench system (Bose Corporation). The tibias were tested in torsion until failure at a rate of 1 deg/sec.

### Data analysis

Myofiber CSA was determined using ImageJ software. The diameter of the fiber was measured at three points along the fiber to get an average cross-sectional area. Myonuclear number was calculated per millimeter length (MN/mm) in the center portion of the myofiber. Myonuclear domain was calculated by dividing myofiber volume [myofiber length (per mm) multiplied by average CSA] by number of myonuclei (per mm). Heat maps were generated using the R package ‘pheatmap’. Results are presented as mean±s.e.m. Statistical significance was determined using Student's *t*-tests for single comparisons and one and two-way ANOVA for multiple comparisons with GraphPad Prism software. *P*<0.05 was considered significant (*P<0.05, **P<0.01, ***P<0.001, ****P<0.0001).

## Supplementary Material

Supplementary information
