# Supplementary material for: Prepubertal skeletal muscle growth requires Pax7-expressing satellite cell-derived myonuclear contribution
Source: Development. 2018 Oct 25;145(20):dev167197. doi: 10.1242/dev.167197 (PMC6215399; doi:10.1242/dev.167197)
Supplement: Supplementary information [file develop-145-167197-s1.pdf]

A **EDL**

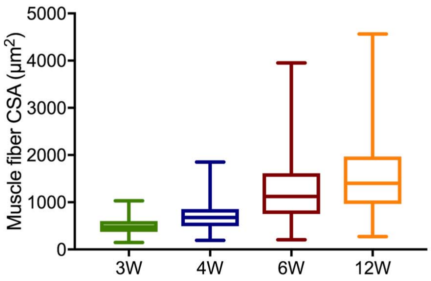

Age (weeks)	Mice	N (Fibers)	Mean CSA	SD	SEM
3W	5	270	469.9	164.1	9.987
4W	5	329	692	260.9	14.38
6W	5	474	1247	659.9	30.31
12W	5	434	1517	757	36.32

B **SOL**

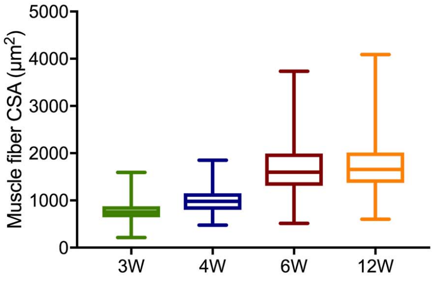

Age (weeks)	Mice	N (Fibers)	Mean CSA	SD	SEM
3W	5	372	766.4	187.6	9.725
4W	5	359	1001	263.3	13.89
6W	5	416	1673	528.9	25.93
12W	5	344	1766	594	32.03

Figure S1. EDL and SOL CSA statistics and box plots

(A and B) Statistics and box plots of EDL (A) and SOL (B) CSA for 3, 4, 6, and 12-week time points.

Development • Supplementary information

A **EDL**

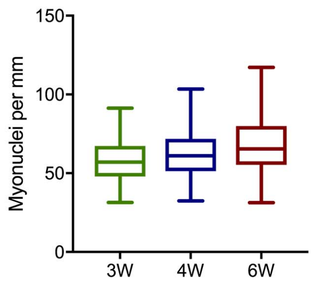

Age (weeks)	Mice	N (Fibers)	Mean Mn/MM	SD	SEM
3W	5	237	57.76	12.04	0.7822
4W	5	246	61.71	13.84	0.8826
6W	5	250	67.84	16.57	1.048

B **SOL**

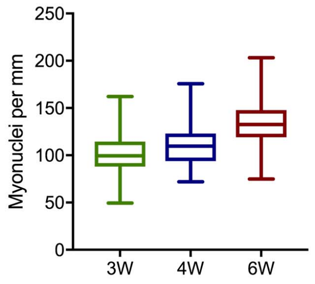

Age (weeks)	Mice	N (Fibers)	Mean Mn/MM	SD	SEM
3W	5	247	101.7	20.25	1.288
4W	5	250	109.9	21.21	1.341
6W	5	250	134.5	23.76	1.503

Figure S2. EDL and SOL myonuclear number statistics and box plots.

(A and B) Statistics and box plots of EDL (A) and SOL (B) myonuclear number (per mm) for 3, 4, and 6-week time points.

Development • Supplementary information

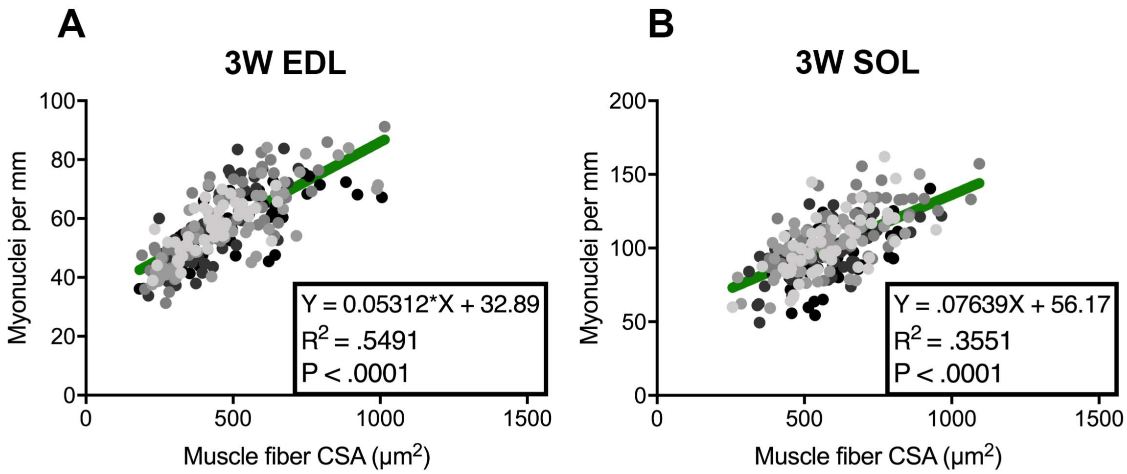

Figure S3. Myonuclear number correlation to CSA at three weeks of age

(A and B) Linear regression analysis displaying correlation between CSA and myonuclear content at 3 week in EDL (A) and SOL (B). Each circle is one myofiber. N= 5 mice. EDL- 237 myofibers and SOL- 250 myofibers.

Development • Supplementary information

A

ECM 6W vs 4W	
Upregulated	Cst3, Mr1, Thrb, Crispld2, Lama5, Prelp, Ecm1, Nav2, Myoc, Lamc3, Col7a1
Downregulated	Postn, Cthrc1, Mfap4, Fbn2, Col2a1, Col11a1, Lox, Col12a1, Acan, Col8a2, Timp1, Col8a1, Eln
	Col1a2, Col14a1, Mmp16, Mfap2, Igf1, Aspn, Olfml2b, Tnc, Col1a1, Matn2, Vcan, Loxl2, Pxdn
	Mfap5, Col3a1, Frem1, Sparc, Cilp, Anxa2, Bgn, Col5a2, Fbn1, Adamts12, Mgp, Itga6, Col6a3
	Col4a, Adamts9, Lamb1, Col6a1, Lama4, Col6a2, Thbs4, Sparcl1, Calr, Col4a2, Nid1, Col15a1, Itgb1

Calcium Signalling 6W vs 4W	
Upregulated	Crebbp, Hdac4, Grina, Hdac5, Ep300, Ryr1, Myh14
Downregulated	Actc1, Acta2, Myl9, Akap5, Casq2, Tpm4, Chrnb1, Casq1, Myl6, Calr, Asph

AMPK Signalling 6W vs 4W	
Upregulated	Ppm1a, Gnas, Pfkfb1, Smarcd3, Prkaa2, Fgfr1, Eef2, Foxo3, Mtor, Pfkm, Tsc1, Ppm1l, Crebbp, Foxo1
	Ulk1, Pik3cd, Acacb, Eef2k, Adra1a, Ep300, Tsc2
Downregulated	Ccna2, Ccnd1, Chrnb1

B

Biological Components GO	Ref List	#	Expected	Fold Enrichment	raw P value	FDR
Developmental process (GO:0032502)	5263	423	243.03	1.74	1.19E-33	9.23E-30
Cellular development process (GO:0048869)	3647	290	168.41	1.72	2.26E-20	1.94E-17
Muscle structure development (GO:0061061)	465	65	21.47	3.03	9.08E-14	2.99E-11
Muscle system process (GO:003012)	225	32	10.39	3.08	1.30E-07	1.06E-05
Skeletal muscle tissue development (GO:0007519)	135	23	6.23	3.69	4.82E-07	3.48E-05
Regulation of muscle adaptation (GO: 0043502)	94	18	4.34	4.15	1.96E-06	1.21E-04

Molcular Function GO	Ref List	#	Expected	Fold Enrichment	raw P value	FDR
Calcium ion binding (GO:0005509)	591	74	27.29	2.71	3.13E-13	1.78E-10
ECM structrual constituent (GO:0005201)	56	21	2.59	8.12	1.08E-11	4.09E-09
Integin binding (GO:0005178)	119	28	5.5	5.1	5.24E-11	1.59E-08
ECM binding (GO:0050840)	60	14	2.77	5.05	3.74E-06	5.87E-04

Cellular Components GO	Ref List	#	Expected	Fold Enrichment	raw P value	FDR
ECM (GO:0044420)	313	63	14.45	4.36	4.59E-20	9.92E-18
ECM component (GO:0044420)	57	20	2.63	7.6	8.84E-11	5.73E-09
Collagen trimer (GO:0005581)	82	24	3.79	6.34	2.81E-11	2.10E-09
Sarcoplasm (GO:0016528)	73	15	3.37	4.45	6.57E-06	2.17E-04
Fibrillar collagen trimer (GO:0005583)	12	6	0.55	10.83	8.56E-05	2.06E-03
Neuromuscular junction (GO:0031594)	73	11	3.37	3.26	1.14E-03	2.02E-02

Figure S4. Relevant differentially expressed genes and GO pathways related to RNA Seq

(A) Up and downregulated genes (at 6 vs 4-week) in Extracellular matrix (GO: 0044420), AMPK Signalling, and Calcium Signaling pathways. DeSeq2 files provided in Supplemental File 2.

(B) Enriched GO terms relevant to muscle development (6 vs 4-week).

Development • Supplementary information

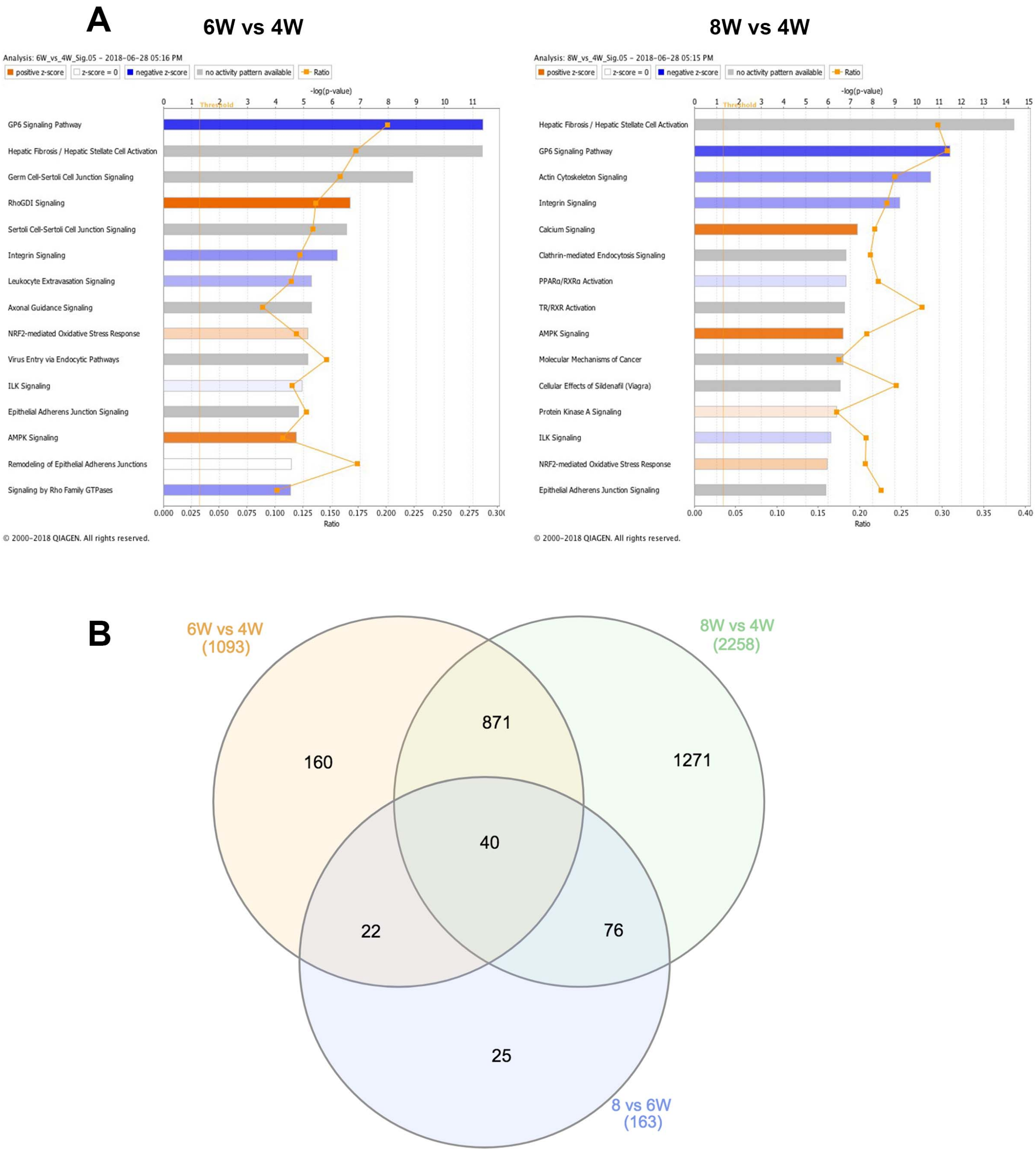

Figure S5. IPA Pathway analysis and Venn diagram related to RNA Seq

(A) Top 15 canonical pathways from IPA analysis of 6 vs 4-week and 8 vs 4-week gastrocnemius muscle. IPA pathway analysis is provided in Supplemental File 2.

(B) Venn diagram displaying differential genes expression at 6 vs 4-week (1093 DE genes), 8 vs 4-week (2258 DE genes), and 8 vs 6-week (163 DE genes) time points.

Development • Supplementary information

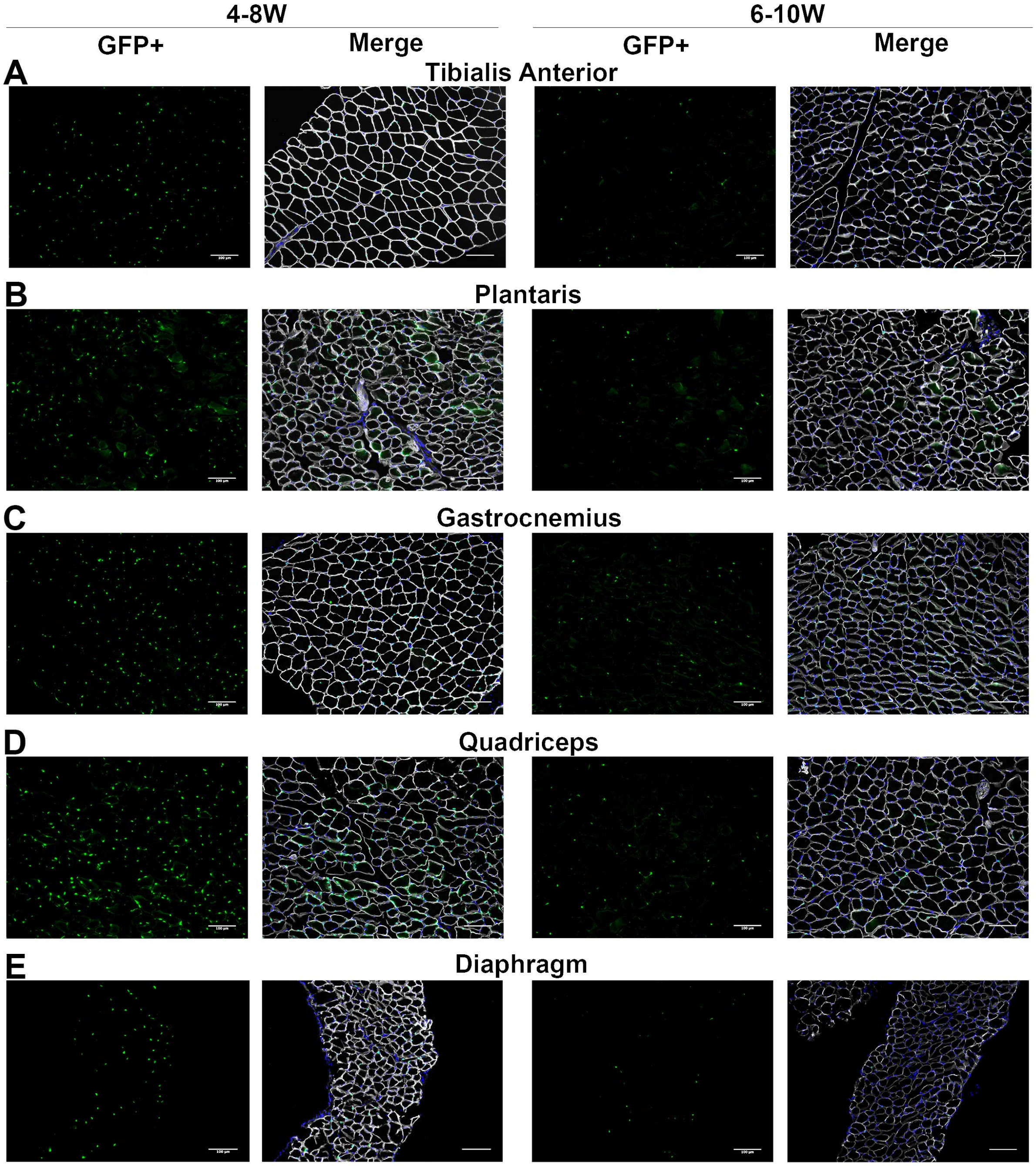

Figure S6. SC contribution to various muscles during prepubertal growth

(A-E) Representative cross-sections of 4-8 and 6-10-week tibialis anterior (A), plantaris (B), gastrocnemius (C), quadriceps (D), and diaphragm (E) muscles following tamoxifen injection (at 4 or 6 weeks) to label SCs and derived myonuclei. Sectioned are stained with GFP (green), DAPI (blue), and laminin (white). Scale bar, 100 μm.

Development • Supplementary information

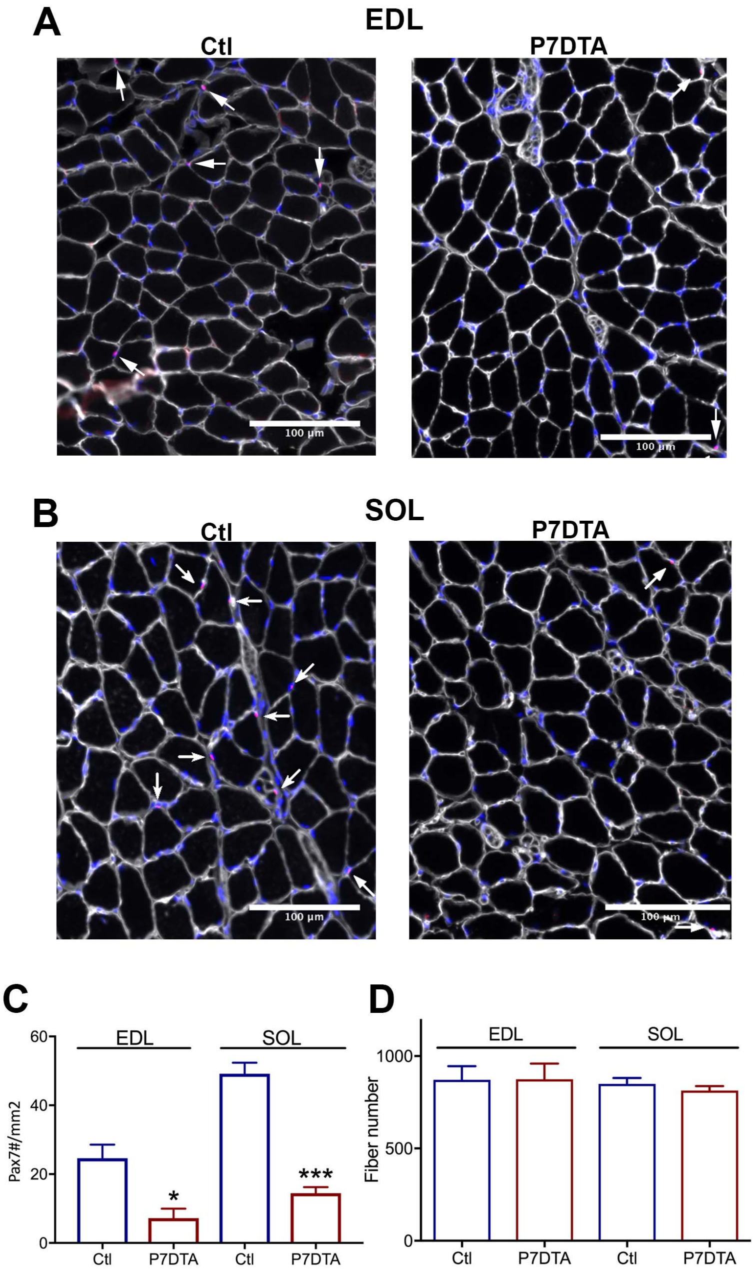

Development • Supplementary information

Figure S7. Significant SC depletion following prepubertal tamoxifen administration

(A and B) Representative Ctl and P7DTA cross-sections for EDL (A) and SOL (B) stained with Pax7 (satellite cells, arrows), DAPI (blue), and laminin (white). Scale bar, 100 µm.

(C) Quantification of Pax7+ SC number in 4W-P7DTA and Ctl EDL and SOL muscles. N= 4 mice per group for EDL and 3-4 per group for SOL. Unpaired t-test.

(D) Quantification of number of fibers per section in P7DTA and Ctl EDL and SOL muscles. N= 5-6 mice per group for EDL and 3-4 per group for SOL.

Development • Supplementary information

Development: doi:10.1242/dev.167197: Supplementary information

A

EDL Myofiber CSA

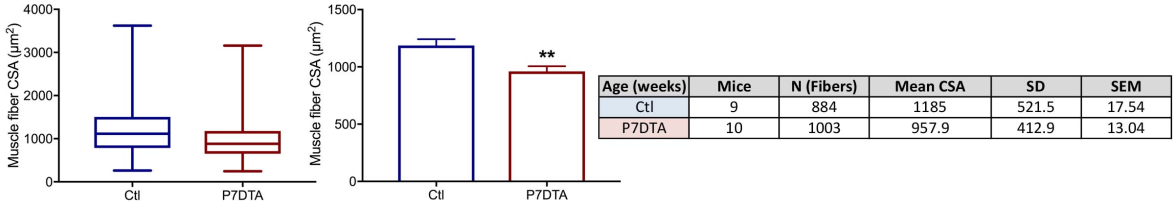

Age (weeks)	Mice	N (Fibers)	Mean CSA	SD	SEM
Ctl	9	884	1185	521.5	17.54
P7DTA	10	1003	957.9	412.9	13.04

B

EDL Myonuclei per mm

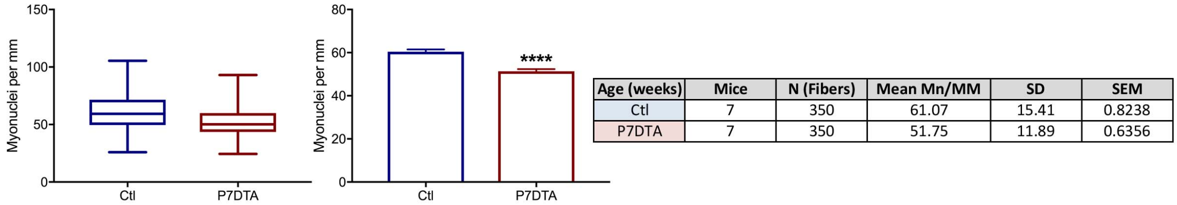

Age (weeks)	Mice	N (Fibers)	Mean Mn/MM	SD	SEM
Ctl	7	350	61.07	15.41	0.8238
P7DTA	7	350	51.75	11.89	0.6356

C

SOL Myofiber CSA

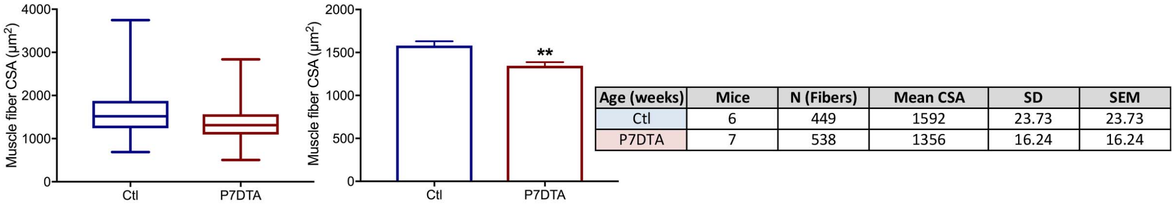

Age (weeks)	Mice	N (Fibers)	Mean CSA	SD	SEM
Ctl	6	449	1592	23.73	23.73
P7DTA	7	538	1356	16.24	16.24

D

SOL Myonuclei per mm

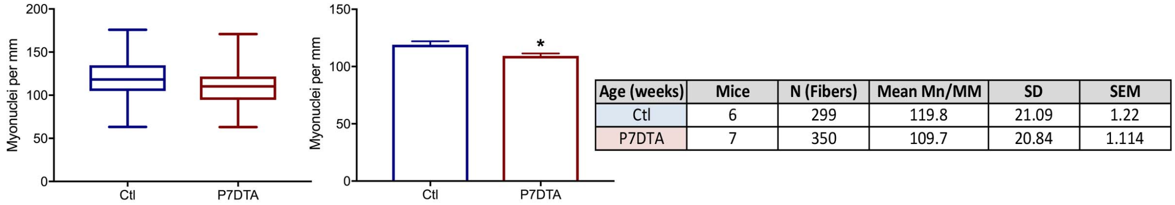

Age (weeks)	Mice	N (Fibers)	Mean Mn/MM	SD	SEM
Ctl	6	299	119.8	21.09	1.22
P7DTA	7	350	109.7	20.84	1.114

Development • Supplementary information

Figure S8. Control vs P7DTA myofiber CSA and myonuclear number statistics (A) Statistics, box plot, and bar graph for Ctl vs P7DTA EDL myofiber CSA. Unpaired t-test.

(B) Statistics, box plot, and bar graph for Ctl vs P7DTA EDL myonuclear number (Mn/mm). Unpaired t-test.

(C) Statistics, box plot, and bar graph for Ctl vs P7DTA SOL myofiber CSA. Unpaired t-test.

(D) Statistics, box plot, and bar graph for Ctl vs P7DTA SOL myonuclear number. Unpaired t-test.

Development • Supplementary information

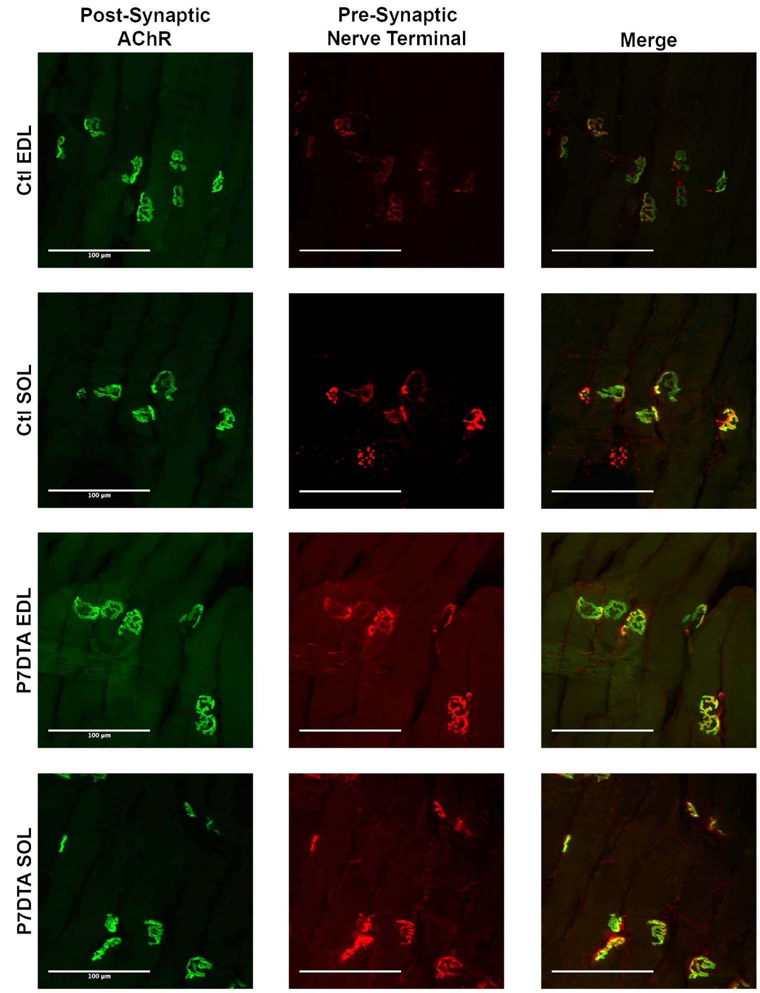

Figure S9. No change in NMJ innervation-integrity with prepubertal SC ablation

(A) Representative images of Ctl and P7DTA neuromuscular junctions in EDL and SOL muscles. Stained for post-synaptic acetylcholine receptor (labeled with Btx, green) and presynaptic nerve terminal (SV2, Syt-2, neurofilament, red). Scale bar, 100 µm. N=1 mouse per group.

Development • Supplementary information

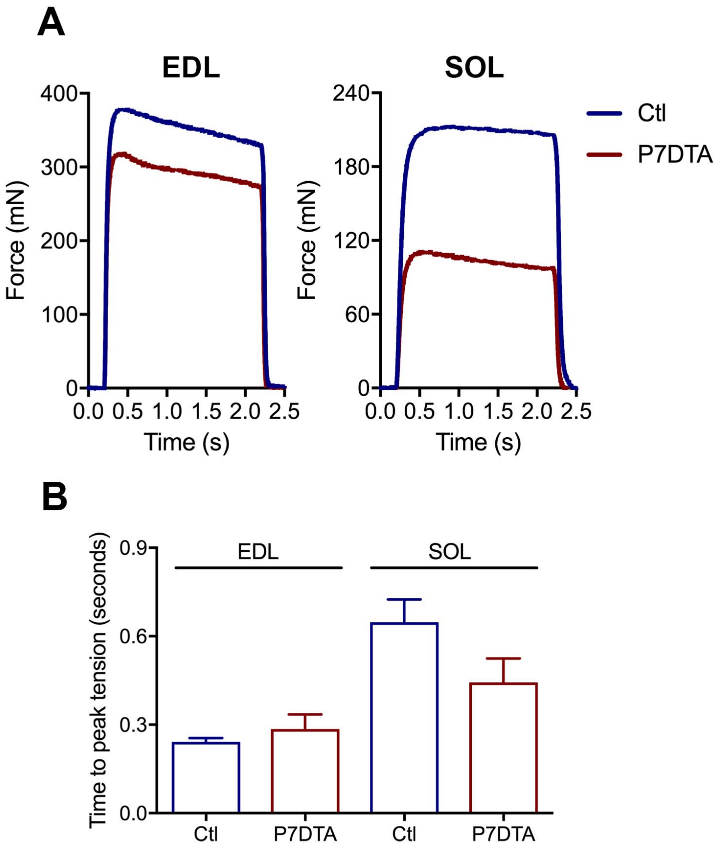

Figure S10. Prepubertal SC ablation results in force generation deficits

(A) Force traces for Ctl and 4W-P7DTA EDL and SOL. Relevant to Figure 9B,C.

(B) Time to peak tension for 4W-P7DTA and Ctl EDL and SOL. Relevant to Figure 9B,C.

Development • Supplementary information

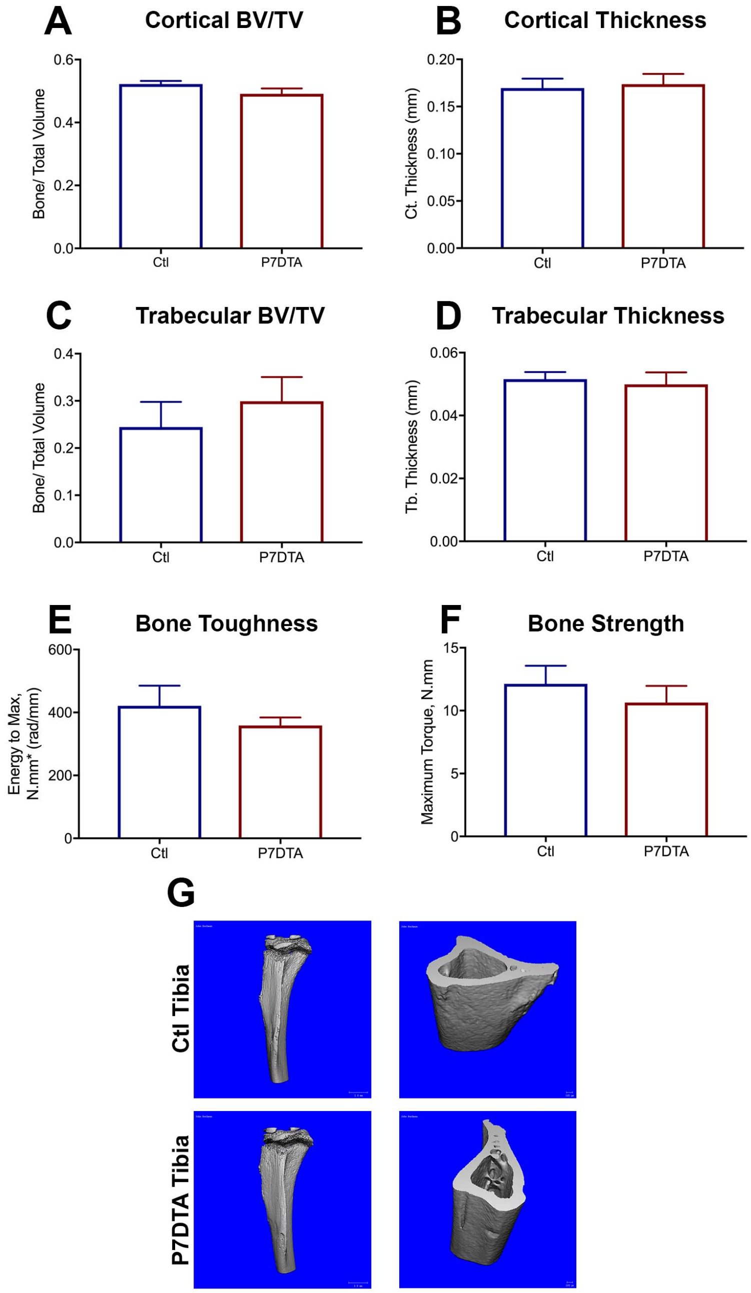

A
Cortical BV/TV
Bone/ Total Volume
0.6
0.4
0.2
0.0
Ctl
P7DTA
B
Cortical Thickness
Ct. Thickness (mm)
0.20
0.15
0.10
0.05
0.00
Ctl
P7DTA
C
Trabecular BV/TV
Bone/ Total Volume
0.4
0.3
0.2
0.1
0.0
Ctl
P7DTA
D
Trabecular Thickness
Tb. Thickness (mm)
0.06
0.04
0.02
0.00
Ctl
P7DTA
E
Bone Toughness
Energy to Max, N.mm* (rad/mm)
600
400
200
0
Ctl
P7DTA
F
Bone Strength
Maximum Torque, N.mm
15
10
5
0
Ctl
P7DTA
G
Ctl Tibia
P7DTA Tibia

Figure S11. No change in tibial architecture or strength with prepubertal SC ablation

(A and B) MicroCT quantitative analysis of tibial cortical bone volume (A) and thickness (B) for Ctl and P7DTA tibias. Cortical bone was analyzed at a 2 mm long section centered at the midshaft of the tibia. N= 4-5 mice per group.

(C and D) MicroCT quantitative analysis for tibial trabecular bone volume (C) and thickness (D). N= 4-5 mice per group

(E and F) Biomechanical torsion testing Ctl and P7DTA tibias. Quantitative analysis of bone toughness and bone strength. N= 4-5 mice per group

(G) Representative microCT images of Ctl and P7DTA tibias.

Development • Supplementary information

Table S1. Statistics related to Figures 1 and 2

Click here to Download Table S1

Table S2. DeSeq2, GO terms, and IPA analysis related to RNA Seq (Fig. 4)

Click here to Download Table S2

Development • Supplementary information

Table S3

qPCR primer list

		**Sequence (5'->3')**
Gapdh	Forward primer	AGGTCGGTGTGAACGGATTTG
	Reverse primer	TGTAGACCATGTACTTGACCTC
Col1a2	Forward primer	TTCTGTGGGTCCTGCTGGGAAA
	Reverse primer	TTGTCACCTCGGATGCCTTGAG
Col3a1	Forward primer	ACGTAGATGAATTGGGATGCAG
	Reverse primer	GGGTTGGGGCAGTCTAGTG
Col6a2	Forward primer	AAGGCCCCATTGGATTCCC
	Reverse primer	CTCCCTTCCGACCATCCGAT
Col11a1	Forward primer	AGGTGGAAAACGAAACGGTG
	Reverse primer	GGAAGAGAAAAGTCAAGGCGA
Hdac4	Forward primer	CACTGCATTTCCAGCGATCC
	Reverse primer	AAGACGGGGTGGTTGTAGG
Loxl2	Forward primer	ATTAACCCCAACTATGAAGTGCC
	Reverse primer	CTGTCTCCTCACTGAAGGCTC

Development • Supplementary information
